# Interventions to Reduce Loneliness in Community-Living Older Adults: a Systematic Review and Meta-analysis

**DOI:** 10.1007/s11606-023-08517-5

**Published:** 2024-01-10

**Authors:** Paul G. Shekelle, Isomi M. Miake-Lye, Meron M. Begashaw, Marika S. Booth, Bethany Myers, Nicole Lowery, William H. Shrank

**Affiliations:** 1 Greater Los Angeles Veterans Affairs Healthcare System, Los Angeles, CA USA; 2 Division of General Internal Medicine & Health Services Research, Department of Medicine, University of California, Los Angeles, Los Angeles, CA USA; 3 General Internal Medicine 111G, West Los Angeles VA Medical Center, Los Angeles, CA USA; 4 Fielding School of Public Health, University of California, Los Angeles, Los Angeles, CA USA; 5 RAND Corporation, Southern California Evidence-Based Practice Center, Santa Monica, CA USA; 6 Louise M. Darling Biomedical Library, University of California Los Angeles, Los Angeles, CA USA; 7 Humana Inc, Lexington, KY USA

**Keywords:** loneliness, social determinants of health, meta-analysis

## Abstract

**Background:**

The problem of loneliness has garnered increased attention from policymakers, payors, and providers due to higher rates during the pandemic, particularly among seniors. Prior systematic reviews have in general not been able to reach conclusions about effectiveness of interventions.

**Methods:**

Computerized databases were searched using broad terms such as “loneliness” or “lonely” or “social isolation” or “social support” from Jan 1, 2011 to June 23, 2021. We reference mined existing systematic reviews for additional and older studies. The Social Interventions Research & Evaluation Network database and Google were searched for gray literature on Feb 4, 2022. Eligible studies were RCTs and observational studies of interventions to reduce loneliness in community-living adults that used a validated loneliness scale; studies from low- or middle-income countries were excluded, and studies were excluded if restricted to populations where all persons had the same disease (such as loneliness in persons with dementia).

**Results:**

A total of 5971 titles were reviewed and 60 studies were included in the analysis, 36 RCTs and 24 observational studies. Eleven RCTs and 5 observational studies provided moderate certainty evidence that group-based treatment was associated with reduced loneliness (standardized mean difference for RCTs =  − 0.27, 95% CI − 0.48, − 0.08). Five RCTs and 5 observational studies provided moderate certainty evidence that internet training was associated with reduced loneliness (standardized mean difference for RCTs =  − 0.22, 95% CI − 0.30, − 0.14). Low certainty evidence suggested that group exercises may be associated with very small reductions in loneliness. Evidence was insufficient to reach conclusions about group-based activities, individual in-person interactions, internet-delivered interventions, and telephone-delivered interventions.

**Discussion:**

Low-to-moderate certainty evidence exists that group-based treatments, internet training, and possibly group exercises are associated with modest reductions in loneliness in community-living older adults. These findings can inform the design of supplemental benefits and the implementation of evidence-based interventions to address loneliness.

**Systematic Review Registration Number:**

PROSPERO (CRD42021272305)

**Supplementary Information:**

The online version contains supplementary material available at 10.1007/s11606-023-08517-5.

## INTRODUCTION

Loneliness is common in community-dwelling seniors, with clear evidence of increasing prevalence during the pandemic.^1^ Numerous studies have found strong associations between loneliness and health outcomes. For example, loneliness or social isolation is associated with a 29% increased risk of heart disease, a 32% increased risk of stroke, and a 50% increased risk of dementia.^2^ Beginning in 2019, the US Centers for Medicare and Medicaid Services (CMS) has allowed Medicare Advantage (MA) plans to target supplemental benefits to beneficiaries’ individual health needs, including more flexibility in the definition of “primarily health-related” and new “non-primarily health-related” benefits.^3^ MA supplemental benefits intended to directly or indirectly address loneliness include in-home support services, group fitness or social classes/memberships, adult day care services, transportation for non-medical needs, and other supports to increase autonomy or functional status.^4^

Evidence to guide which interventions to offer is needed. In recognition of these needs, the US National Academies of Sciences, Engineering, and Medicine (NASEM) released a report in 2020 about social isolation and loneliness in older adults.^2^ As part of this, the Committee reviewed the literature on interventions, citing 7 existing “large scale reviews”.^5–11^ From these and data from some individual studies, the Committee concluded that “a variety of interventions have been proposed….however there is not enough evidence to identify the most effective interventions”; and “many intervention studies do not use a validated tool.” Until recently prior systematic reviews have either been narrative,^5–9, 11^ or if meta-analytic have been very broad in population (for example, statistically combining studies of interventions for children in the third grade with interventions for older adults living in nursing homes),^10^ or narrowly focused on intervention, specifically computer and internet use or information and communication technology,^12–16^ or were a scoping review.^17, 18^ To address these limitations, our goal was to conduct a systematic review and meta-analysis of diverse interventions to reduce loneliness in a more homogeneous population, namely older community-living adults, with loneliness being measured with a validated measure.

## METHODS

This review is reported using *Preferred Reporting Items for Systematic Reviews and Meta-analyses* criteria. The funder participated in setting the scope of the review and the interpretation of the results. The public was not involved.

### Data Sources and Searches

Interventions to reduce loneliness have been the subject of numerous prior reviews. Therefore, we adopted a 3-phase search strategy: search the references (reference mine) in existing systematic reviews; new searches for published literature; and then gray literature searches. We started with the seven reviews^5–11^ cited in the NASEM report.^2^ To this, we added the rapid review by the US Agency for Healthcare Research and Quality,^19^ and six other systematic reviews identified on a preliminary search.^12, 16, 20–23^ We reference mined all these reviews. We then used articles contained in these reviews to construct a search strategy that would find more articles similarly indexed. We used this strategy to query Ovid Medline and the Cochrane Library from January 1, 2011, to June 23, 2021, using terms such as “loneliness or lonely or friendship or solitude or aloneness” or “social support or social isolation” (see eTable 1 for the full search strategy). To this, we then added a search of the Social Interventions Research & Evaluation Network (SIREN) database and 7 searches on Google for additional published and gray literature (searched on February 4, 2022). Finally, we searched for studies via reference mining which included original research studies and expert consultation, with no restriction on publication date.

### Study Selection

Two authors (PGS and IML) independently screened titles, abstracts, and full texts, with disagreements reconciled through team discussion. Studies were initially eligible if they (1) were focused on community-living adults; (2) had an intervention whose intent was to reduce loneliness; and (3) reported loneliness outcomes. Later, we added an additional criterion, that the duration of follow-up had be greater than 4 weeks. Randomized and observational studies (but not cross-sectional or case-control studies) were included. Studies in children or adolescents were excluded. Since there is not a bright line for what constitutes “older” adults, we did not enforce an age threshold other than adults, but almost all studies were about adults older than age 50. Studies of adults living in nursing homes were excluded, but adults living independently in congregate living facilities were included. Studies where the target population were selected because of some particular health condition, such as blindness^24^ or dementia,^25^ were excluded because we judged that generalizing to other populations would be difficult. Due to the perceived importance of context in who is lonely and the availability of potential interventions to reduce the risk of loneliness, we excluded studies from low- and middle-income countries.^26^ We excluded studies that were not originally designed as interventions to reduce loneliness, such as a study of Meals On Wheels,^27^ because we judged these to be at high risk for selective outcome reporting. After performing the initial screening, and when considering the meta-analysis, we restricted eligibility to studies that used one of two validated loneliness scales described in the NASEM report as measuring essentially the same aspects of loneliness: the UCLA scale^28^ or the deJong Gierveld scale,^29^ which together accounted for about 84% of all studies reporting loneliness outcomes. We also included 2 articles that used outcome measures that provided evidence that they were correlated at least 70% with either the UCLA scale or the deJong Gierveld scale.^30, 31^ Lastly, we excluded 8 studies who met all the other eligibility criteria because they did not report quantitative data sufficient to be used in the meta-analysis (for example, a study only reported “no differences” in loneliness between the pre- and post-test evaluation).^32^

### Data Extraction and Quality Assessment

Data elements extracted in duplicate included study design, intervention characteristics, population characteristics, and follow-up. We assessed whether enrolled populations were at increased risk of loneliness via selection due to life circumstance (such as bereavement) or by using a tool screening for loneliness. For risk of bias, we used the Cochrane Risk of Bias Tool,^33^ the Risk of Bias in Non-Randomized Studies of Interventions tool,^34^ or a modification of the NIH tool for Pre-Post studies.^35^ Data on outcomes were extracted by the statistician and checked by a second author.

### Data Synthesis and Grading

**Figure 1:**
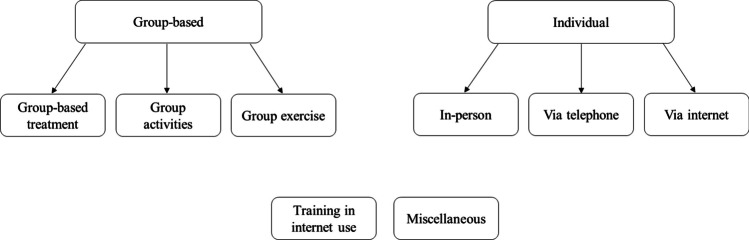
Categories of loneliness interventions.

There is no standard method for grouping interventions to reduce loneliness into categories sufficiently similar to support meta-analysis. Ideally, we would like interventions within a group to be identical, for example, the way pharmaceutical interventions can be considered to be identical. Unfortunately, since only two interventions were the subject of more than one study,^36–40^ this would result in four dozen different categories, nearly all with only a single study in it, and thus, no meta-analysis would be possible. The most common way of grouping studies in prior systematic reviews has been whether interventions were delivered via a “group” or “one-to-one.”^18^ We adopted this scheme in general, but additionally separated “group” interventions into those that included some kind of mental or cognitive treatment (such as “participatory group-based care management,”^41^ “group-based educational, cognitive, and social support,”^42^ or “discussions guided by Self-management of Well-being theory”^43^) versus those that involved only an activity (such as group-based dance, or singing in a chorus^44^) versus studies that were only group-based exercises (Tai Chi,^45^ aerobics,^46^ or a structured supervised exercise program).^47^ For “one-to-one” (which we call “individual in-person interactions”), we separated these into whether they were in-person, or delivered over the internet, or by telephone (see Fig. 1). We kept as its own category 10 studies of interventions that trained older adults in how to use the internet and/or social media. Lastly, there remained 8 studies of interventions that did not fit into any of the above categories, they being an eclectic mix such as volunteering as a foster grandparent, writing about one’s life experiences, or a computer-tailored intervention designed to stimulate cognitive function and increase physical activity.

The majority of studies reported continuous outcomes so we report the results as standardized mean differences. We kept studies using a randomized design separate from observational studies. Each has its relative strengths and weaknesses in terms of internal validity and external generalizability. When sufficient studies existed (three or more) within each intervention and study design category, we pooled them using a random effects meta-analysis. We use the Hartung-Knapp-Sidik-Jonkman^48–51^ method for our random effects meta-analysis. This method has been preferred when the number of studies pooled is small and when there is evidence of heterogeneity.^51^ We used the *I*^2^ statistic^52^ to assess the level of heterogeneity. Egger’s regression asymmetry test^53^ and Begg’s rank correlation^54^ were used to examine publication bias. All analyses were conducted in R.4.0.2 using the metafor package.

We rated certainty of the evidence using the Grading of Recommendations Assessment, Development, and Evaluation (GRADE) system.^55^

## RESULTS

After screening 5971 titles and adding 175 full text articles from reference mining existing systematic reviews and 16 studies from our gray literature search, we identified 88 potentially eligible studies (see eFigure 1). After rejecting 14 studies because they did not use either the UCLA or the DeJong Gierveld loneliness scale^56–69^ or a scale shown to be highly correlated with either, five studies for short follow-up time,^70–74^ and a further eight studies because of insufficient data (see eTable 2),^32, 75–82^ and one study because we judged its 4% follow-up rate to be so low that no valid conclusions could be drawn,^83^ there were 60 full text articles that were included in our quantitative analysis (see eTable 3 for a list of excluded studies).

### Description of the Evidence

Of the 60 studies, 36 were randomized trials and 24 were observational studies. All but four studies were either restricted to adults that were at least aged 50 or older or had a mean age that was over age 50.^39, 40, 84, 85^ Nineteen studies were done in the USA, twelve studies were done in the Netherlands, five studies were done in the UK, four studies each were done in Canada and Australia, and the remainder came from Israel, Spain, Sweden, Finland, Italy, Switzerland, Hong Kong, and Japan.

### Risk of Bias Assessment

The assessments of the Cochrane Risk of Bias criteria for the randomized trials are in eTable 4 and of the Risk Of Bias in Non-randomized Studies of Interventions criteria in eTable 5; and the adaptation of the NIH tool is in eTable 6. These types of interventions are essentially impossible to blind, and the assessments reflect this. However, we did not place much weight on blinding when assessing a study’s limitations. Since studies were selected only if they were intended to reduce loneliness and used a validated scale, all studies were judged to be at low risk of bias for selective reporting and in measurement of the outcome. Thus, studies were discriminated primarily on design, and then on details of how subjects were selected and offered different treatments, the attrition rate, and the sample size.

### Group-Based Treatment

**Figure 2:**
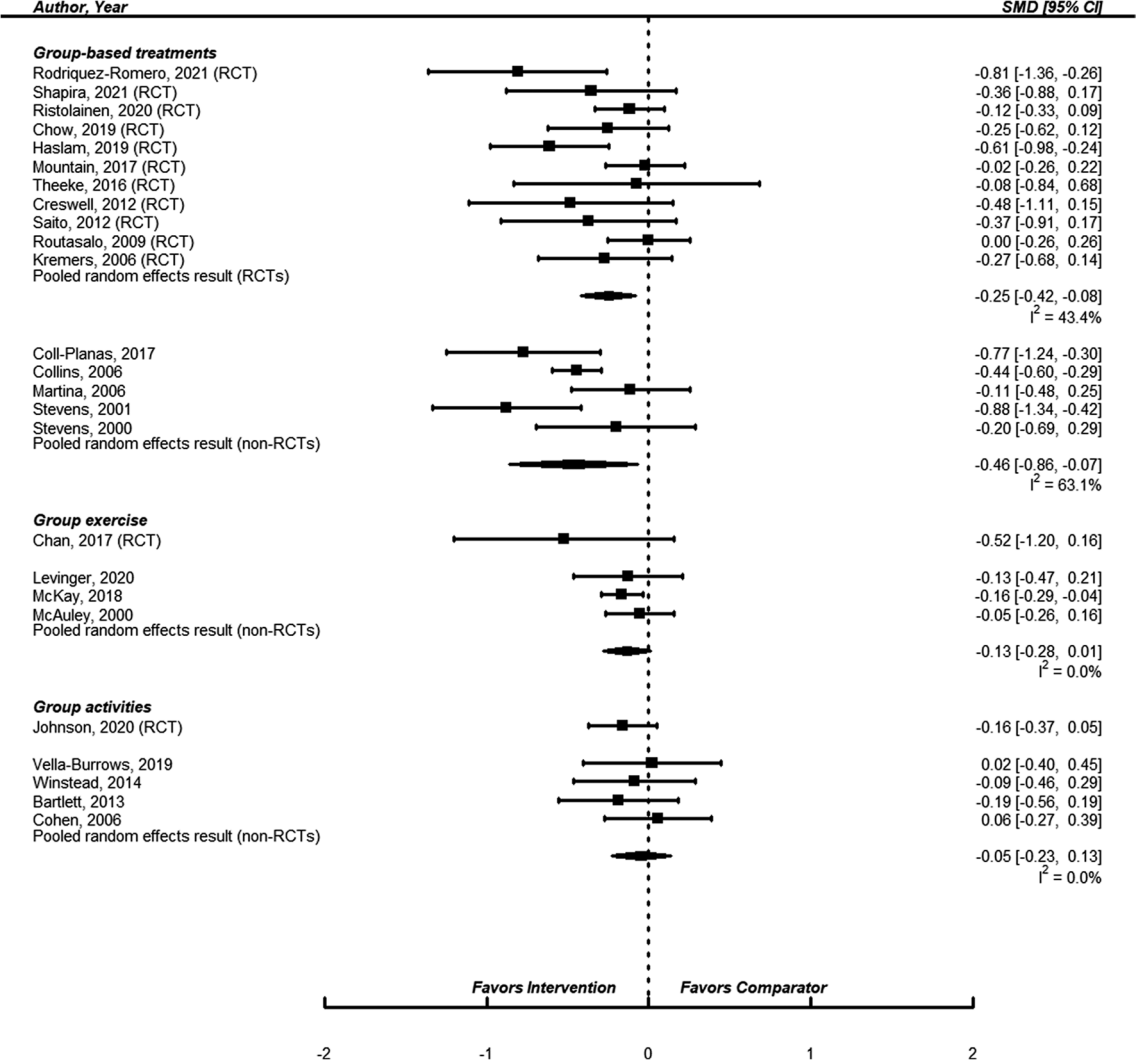
Forest plot of group-based interventions to reduce loneliness.

We identified 16 studies of group-based treatment, 11 randomized trials^41–43, 84, 86–92^ and 5 observational studies.^36–38, 93, 94^ Twelve studies enrolled populations selected for increased risk of loneliness, and four studies had unselected populations. In all but one study enrollment criteria or the mean age of subjects was 50 years of age or older. The content of the treatment was heterogeneous across studies but was in general based on psychological theories or principles (such as cognitive behavioral therapy, mindfulness, participatory group-based care management, discussion topics that were “based on the social cohesion approach of social capital theory,” feminism, “re-evaluation counseling,” etc.) (see Table 1). In some interventions, there were also practical topics about day-to-day matters, like nutrition and food, and healthy aging. A few also included exercises (yoga, group exercises). What all interventions had in common was they involved bringing together small groups of subjects for regular group sessions, usually for 2–4 months in duration, and the sessions were in general led by a trained moderator. The random effects pooled estimate of effect for the 11 RCTs or the 5 observational studies both showed that there was less loneliness in the group-based treatment subjects (standardized mean difference of 11 RCTs: − 0.25 (95% confidence interval − 0.42, − 0.08); standardized mean difference of 5 observational studies: − 0.46 (95% confidence interval − 0.86, − 0.07)) (see Fig. 2). One test assessing the possibility of publication bias was statistically significant (Egger’s regression test for RCT pooled result = 0.01); however, Begg’s rank correlation test for the RCT result and neither test for the observational study results were statistically significant.

**Table 1:** Details of Group-Based Treatment Studies

Study/country/study design	Age of subjects	Selected for increased loneliness risk?	Topics for group discussion	Duration of group meetings	Moderator
Chow, 2019^92^ Hong Kong RCT	Mean age = 74	Yes	Dual-process model of coping with bereavement consisting of (1) loss-oriented coping; (2) restoration-oriented coping; and (3) oscillation	8 sessions with 16-week follow-up	Experienced bereavement counselors
Coll-Planas, 2017^93^ Spain Pre-post	≥ 60	Yes	“Based on the social cohesion approach of social capital theory”	Weekly for 15 weeks	Health and social care professionals
Collins, 2006^94^ USA Pre-post	Mean age = 73	No	Lessons on nutrition and food, personal safety, general wellness, financial strategies, productive aging, etc	Weekly for 15 weeks	Paraprofessional, volunteers and peer educators
Creswell, 2012^89^ USA RCT	*55–85*	No	Mindfulness-based stress reduction	8 weekly 2-h sessions and a day-long retreat	Trained clinicians
Haslam, 2019^84^ Australia RCT	Mean age = 31	Yes	Group 4 Health, a unique theory-based strategy for addressing the lack of belonging	5 modules over 2 months	Psychologist
Kremers, 2006^43^ Netherlands RCT	≥ 50	Yes	Discussions guided by Self-Management of Well-being theory	Weekly sessions for 6 weeks	“Female leaders”
Martina, 2006^36^ Netherlands Non-randomized study	≥ 55	Yes	Friendship Enrichment Program, based on the principles of feminism, and a self-help method called re-evaluation counseling	Weekly sessions for 12 weeks	Not reported
Mountain, 2017^88^ England RCT	≥ 65	No	Lifestyle Matters, a manualized intervention discussing a wide range of topics	Weekly sessions for 4 months	National Health Service or social care staff who were trained
Ristolainen, 2020^41^ Finland RCT	≥ 65	Yes	“Participatory group-based care management,” social support, counseling, activities	5 sessions over 6 months	Care manager and researcher
Rodriguez-Romero, 2021^86^ Spain RCT	≥ 65	Yes	Mindfulness, healthy nutrition, yoga, needs of aging, going to a movie, etc	18 sessions over 6 months	Various
Routasalo, 2009^90^ Finland RCT	≥ 75	Yes	Art and inspiring activities, group exercises and discussion, and therapeutic writing and group therapy	Weekly sessions for 3 months	Registered nurse, occupational therapist, or physical therapist
Saito, 2012^42^ Japan RCT	Mean Age = 73	Yes	Group-based educational, cognitive, and social support program	4 sessions over 6 weeks	“Member of the community experienced in leading group activities”
Shapira, 2021^87^ Israel RCT	≥ 65	No	CBT^*^, social interaction, mindfulness	Twice weekly for 3 months	Clinical social worker, over Zoom
Stevens, 2000^38^ Netherlands Pre-post	54–80	Yes	Friendship Enrichment Program (see Martina, above)	Weekly sessions for 12 weeks	Not reported
Stevens, 2001^37^ Netherlands Pre-post	54–83	Yes	Friendship Enrichment Program (see Martina, above)	Weekly sessions for 12 weeks	Not reported
Theeke, 2016^91^ USA RCT	Mean age = 75	Yes	LISTEN, a cognitive behavioral intervention for loneliness	2-h weekly sessions for 5 weeks, 12 week follow-up	Trained interventionalists

^*^Cognitive behavioral therapy

### Group-Based Exercises

We identified 4 studies of group-based exercises, 1 RCT^45^ and 3 observational studies.^46, 47, 95^ All were of subjects unselected for increased risk of loneliness. All studies enrolled subjects who were 60 years or older or whose mean age was greater than 60. The exercises in these 4 studies consisted of Tai Chi, a “structured supervised exercise program,” a personalized plan and group sessions to sustain the plan, and aerobic exercises, stretching and toning (see Table 2). The one RCT reported less loneliness in the exercise group (standardized mean difference of − 0.52, 95% confidence interval − 1.20, 0.16). The random effects pooled estimate of effect for the 3 observational studies showed there was less loneliness in the exercise group (standardized mean difference − 0.13 (95% confidence interval − 0.28, 0.01)) (see Fig. 2). There was no evidence of publication bias.

**Table 2:** Details of Group Exercise Studies

Study/country/study design	Age of subjects	Selected for increased risk of loneliness?	Details of exercises	Duration of the exercise program
Chan, 2017^45^ Hong Kong RCT	≥ 60	No	Tai Chi	3 months
Levinger, 2020^47^ Australia Pre-post	≥ 60	No	Structured supervised exercise program led by qualified exercise instructor	12 weeks
McAuley, 2000^46^ USA Pre-post	Mean age = 66	No	Aerobic exercises, stretching and toning	6 months
McKay, 2018^95^ Canada Pre-post	≥ 60	No	Consultation with activity coaches to set goals and personalized plan; group sessions on developing and sustaining the activity plan	3 months

### Internet Training

**Figure 3:**
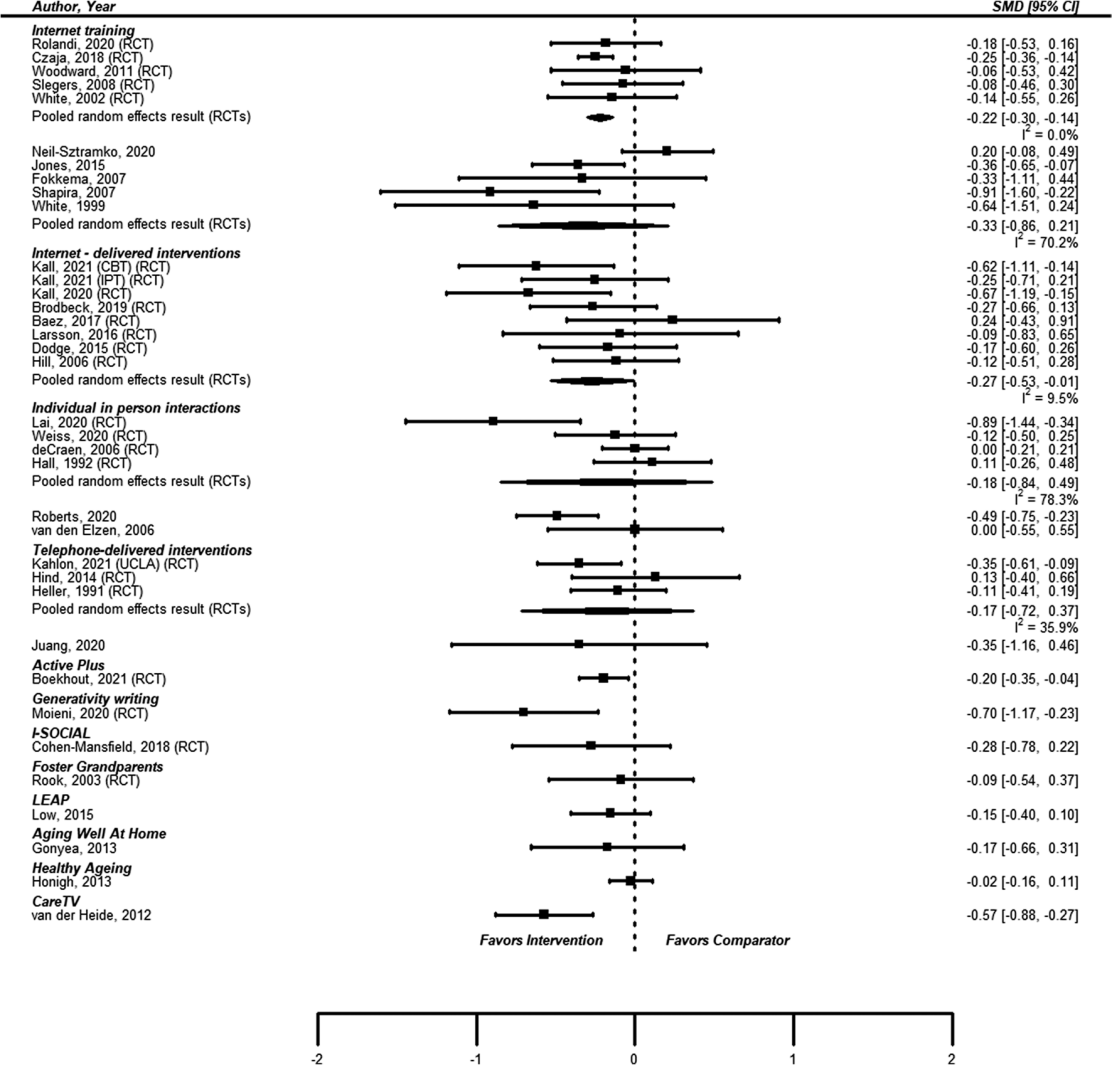
Forest plot of miscellaneous interventions to reduce loneliness.

We identified 10 studies of internet training, 5 randomized trials^40, 96–100^ and 5 observational studies.^101–105^ Only 1 study enrolled a population selected to be at increased risk of loneliness.^97^ In all studies, the subjects were restricted to or had a mean age of 60 years or older. In older studies, the training consisted of basic computer skills, internet use, email competency, etc. More recent studies included training regarding social media, photographs, and video chat applications. For studies with a concurrent comparison group, comparators received no intervention or activities other than internet training (like sewing or painting) or, in one study, a binder with the same printed content as in the training (see Table 3). The random effects pooled estimates of effect for the 5 RCTs and for the 5 observational studies showed less loneliness in the internet training group (standardized mean difference for 5 RCT studies =  − 0.22 (95% confidence interval − 0.30, − 0.14); standardized mean difference for 5 observational studies =  − 0.33 (95% confidence interval − 0.86, 0.21)) (see Fig. 3). There was no evidence of publication bias.

**Table 3:** Details of Internet Training Studies

Study	Age	Selected	Training	Mode and duration of intervention
Czaja, 2018^97^ USA RCT	≥ 65	Yes	Intervention: Purpose-built computer systems for older adults -Computer/Monitor/Printer -Purpose-built software -Internet access -Online help -Training sessions Control: Binder with similar content	Home visit, check-in calls 12 months
Fokkema, 2007^103^ Netherlands Pre-post	“Seniors” mean age = 66	No	-Computer equipment -Internet access -Internet use -Email	5 2-h sessions delivered in their homes
Jones, 2015^102^ England Pre-post	Mean age = 63	No	-Basic computer use -Internet -Skype or Facetime -Online shopping, news and entertainment Some subjects received computers and internet access	12 h of support either in groups or one-on-one sessions
Neil-Sztramko, 2020^101^ Canada Pre-post	Mean age = 76	No	-Use of tablet computer -Learning basic features, locating apps -Use of specific apps -Internet, Photos, Email	2-h sessions weekly over 6 weeks
Rolandi, 2020^96^ Italy RCT	Mean age = 81	No	Intervention: -Smart phone use -Facebook and WhatsApp use -Privacy rules, fraud risk prevention Control: Waiting lists, Lifestyle education	5 group sessions twice a week and face-to-face individual tutoring available
Shapira, 2007^104^ Israel Non-randomized study	Mean age = 80	No	Intervention: -Computer access -Email, Web browsing, forums and virtual communities Control: Painting, sewing, needlework, ceramics	15 weeks
Slegers, 2008^99^ Netherlands RCT	64–75	No	Intervention: -Computer, basics of computer use -Internet applications, searches, email, browser Control: No training or computer	3–4 h of training sessions over 2 weeks
White, 1999^105^ USA Non-randomized study	Mean age = 79	No	-Access to computer -Email, internet use -Help desk support Control: Waiting list	9 h of instruction
White, 2002^100^ USA RCT	Mean age = 72	No	Intervention: -Internet training -Email, browsing Control: Waiting list	9 h of group training over 2 weeks
Woodward, 2011^98^ USA RCT	≥ 60	No	Intervention: -Basics of computer use -Blogging, photos -Use of voice and video over the internet -Favorite senior sites, Genealogy -Downloading music and books -Greeting cards -Online discussion sites -and more Control: -No training	Every 2 weeks for 11 weeks

### Internet-Delivered Interventions

We identified 7 studies of internet-delivered interventions, all of which were RCTs.^39, 40, 85, 106–109^ Five of the seven studies enrolled populations selected to be at increased risk for loneliness. Four of the studies had mean ages of enrolled subjects less than 65 years.^39, 40, 85, 106^ Three studies assessed internet-delivered cognitive behavioral therapy (CBT);^39, 40, 106^ the remainder had content unique to the study (see eTable 7). The random effects pooled estimate of effect for the 7 RCTs (using the CBT arm of the study by Kall 2021 as the intervention) showed less loneliness in the intervention groups (standardized mean difference =  − 0.27 (95% CI − 0.53, − 0.01)). There was no evidence of publication bias. The point estimates of effect for the 3 RCTs of CBT were higher than the point estimates for any of the other internet-delivered interventions.

### Results for Other Interventions, Head-to-Head Studies, and Sensitivity Analyses

#### Other interventions

Six studies of individual in-person interactions^110,111–115^, 4 studies of telephone-based interventions (all 4 of which were individual one-on-one, one study also included group phone calls),^30,116–118^ and 8 studies unable to be classified with any of the other groups^119,120–126^ provided signals that they may have associations with less loneliness, but individually and meta-analytically their results were not as strong as the four interventions discussed in detail. One RCT^31^ and four observational studies^44,127–129^ of group activities provided no evidence of an effect on loneliness. See Figs. 2 and 3, and eTables 8–11 for additional details of these studies.

#### Head-to-head studies

We identified one study that compared different interventions for loneliness. This study randomized 170 lonely adults with mean age of 47.5 years, and of whom, 76% were women to either internet-delivered cognitive behavioral therapy (iCBT) or interpersonal psychotherapy (iIPT) or a wait-list control. At 4 months, with 61 subjects lost to follow-up, there was a greater reduction in loneliness as measured by the UCLA scale for subjects treated with iCBT than with iIPT.^39^

#### Assessing for effect modifiers

An attempt at meta-regression to assess the potential for effect modification due to selection of subjects at increased risk of loneliness was not possible due to very strong correlations between type of intervention and such selection.

#### Studies excluded due to outcome measure 

We did not include 14 studies in our analysis due to their outcomes measure being something other than the UCLA loneliness scale or the deJong Gierveld loneliness scale. Eight of these studies used outcome measures that were dichotomous (e.g., “Are you lonely?”) or categorical,^60–65, 67, 69^ for which we were able to calculate an odds ratio for six.^60–62, 64, 67, 69^ This included one study that used the deJong Gierveld scale but then analyzed it as a dichotomous outcome.^67^ The remaining six studies^56–59, 66, 68^ used scales for which we calculated an SMD. Appendix eTable 12 gives details about these studies and shows forest plots of loneliness outcomes, by the type of intervention. Inclusion of any of these studies would not materially change any of our conclusions.

### Certainty of Evidence

We judged the certainty of evidence that group-based treatment is associated with lower levels of loneliness as moderate, reduced from high due to serious inconsistency. We judged the certainty of evidence that internet training is associated with lower levels of loneliness as moderate, again reduced from high due to serious inconsistency. While we did not consider the observational study evidence in either case when assessing the certainty of evidence using GRADE, in both cases, we considered the agreement in pooled results between data from RCTs and from observational studies to strengthen the conclusion. We judged the evidence that group-based exercise therapy is associated with lower levels of loneliness as low, reduced due to serious concerns about study risk of bias and serious imprecision (eTable 13). Although the pooled estimate of effect for internet-delivered interventions was similar to the pooled estimate of effect for the above interventions, we judged the certainty of evidence as very low due to increased heterogeneity in the content of interventions and increased indirectness in the population. All other potential associations are judged as being very low certainty.

## DISCUSSION

The principal finding of this systematic review is that there are interventions associated with lower levels of loneliness in community-living older adults, in specific group-based treatment and internet/social media training. Group-based exercises may possibly also be associated with lower levels of loneliness. The effect size for association for any of these interventions is modest, using standard yardsticks to assess the meaning of an effect size. Nevertheless, to put this in perspective, the size of this effect on loneliness is roughly similar to the lower end of pooled estimates of the effect of oral antidiabetic agents on hemoglobin A1c levels,^130^ which is not an effect that is clinically insignificant.

Strengths of our review are that we included more RCTs and more studies in general than prior reviews, the classification of interventions into categories based on content and delivery, and the comparison and contrast of results from RCTs with results from observational studies, which tend to support each other. The key limitations to this review are that we may not have identified all of the relevant evidence, and residual heterogeneity among the evidence we did find. Regarding the former point, any potentially eligible studies we missed must in turn have been missed by all systematic reviews we reference mined and missed by the 2020 National Academies of Sciences, Engineering, and Medicine report, and missed by the recent comprehensive review by Hoang and colleagues.^131^ Unpublished studies also fall within the category of missing evidence, and while we did not find definitive evidence of the presence of publication bias, the statistical tests for it are known to be underpowered and so we always assume that some unpublished studies must exist. Their effect on our results is speculative. Furthermore, it is likely that as a result of the pandemic more loneliness research has been recently published and will be published in the future. Regarding residual heterogeneity, there is certainly heterogeneity among included studies within our intervention categories—without tolerating some heterogeneity, no pooled analysis would have been possible. We grouped studies using variables similar to those used by other review authors,^18^ but acknowledge that within a single intervention category, such as group-based treatment, there are potential differences in effectiveness between interventions. Whether differences in individual study outcomes are due to differences in the content of their group treatment, or difference in the populations studied, or just randomness, is impossible at this point to tell. Nevertheless, the demonstration that across a possibly heterogeneous collection of group-based treatment studies there is a significant association with less loneliness should provide the impetus for more precise studies trying to identify the most effective components and how this may differ between different populations, what dosage is most effective, and for how long it may last.

Our results go beyond and extend the findings of prior systematic reviews and the conclusions of the NASEM report. In general, prior reviews have been unable to reach conclusions about effectiveness, being narrative and citing the paucity and methodologic limitations of the primary studies, with the concomitant call for more and better research. The NASEM report mirrored these conclusions. Recently, as our review process was being completed, there was published another review of interventions for loneliness in older adults. While there are some similarities between the review by Hoang and colleagues^131^ and our review—for example, both are focused on older adults, both have meta-analytic results for community-living subjects—there are also important differences (see eTable 14). While in the review by Hoang and colleagues all their conclusions were judged as very low certainty evidence, such was not the case in our review.

We may speculate about why some interventions seemed to be more effective than others. While acknowledging that there are no head-to-head trials to prove the superiority of one class of interventions over another, our results suggest the framing of the intervention matters, possibly as a mechanism to reduce the stress, hypervigilance, and vulnerability experienced by states of loneliness.^132^ Group treatments may be more effective than group activities like exercise because in the former people are forced by the treatment itself to verbally interact with their fellow subjects and may find the concept of a treatment more reassuring than an activity. In group activities, a person could engage less fully, and the perceptions of vulnerability that created the subjective experience of loneliness may create additional barriers to full participation. Internet training may work better than internet-delivered therapies because internet training fosters more agency among lonely patients, as well as facilitating engagement in social media and self-directed learning as compared with an internet therapy that feels more scripted and more like “work.” Again, the framing of the intervention may enhance engagement and deeper participation, the former engenders more enthusiasm while the latter may feel like a chore.

These findings have important implications for payors, providers, and policymakers. For policymakers, these results reaffirm the importance of flexibility in offering programs (either as benefits or directly provided) to address health-related social needs such as loneliness. While further research is needed to assess the relationship between interventions to improve loneliness and health outcomes (such as mental or physical health) or costs, these findings underscore the promise in these interventions to address the underlying social need. Additional studies are needed to better understand whether specific interventions are more effective in certain population subgroups, such as those at increased risk of loneliness, which will improve overall effectiveness of these efforts.

## CONCLUSIONS

This systematic review and meta-analysis found moderate certainty evidence that two types of interventions—group-based treatment and training to use the internet (including social media) are associated with lower levels of loneliness in populations that are mainly community-living older adults. These findings can inform the design of supplemental benefits or programs and the implementation of evidence-based interventions to address loneliness.

### Supplementary Information

Below is the link to the electronic supplementary material.

Supplementary file1 (DOCX 489 KB)

Supplementary file2 (DOCX 32 KB)

## Data Availability

All data used in the analysis are presented in the article or the supplemental material.
