# Supplementary material for: Interventions to Reduce Loneliness in Community-Living Older Adults: a Systematic Review and Meta-analysis
Source: J Gen Intern Med. 2024 Jan 10;39(6):1015–28. doi: 10.1007/s11606-023-08517-5 (PMC11074098; doi:10.1007/s11606-023-08517-5)
Supplement: Supplementary file 1 — Supplementary file1 (DOCX 489 KB) [file 11606_2023_8517_MOESM1_ESM.docx]

**Appendices**

**eTable 1. Search Strategy**

**DATABASE SEARCHED & TIME PERIOD COVERED**

Ovid MEDLINE – limited to January 1, 2011 – June 23, 2021

((exp "Aging"/ or ("older adults" or elder* or senior* or geriatric or aging or ageing or retire* or "old age" or "older people" or "older women" or "older men").ti,ab,kw,bt.) and ("clinical trial".pt. or exp "program evaluation"/ or exp "epidemiologic studies"/ or (intervention* or random* or program* or trial or evaluat* or implement*).ti,bt. or (pilot* or "control group" or "quasi-experimental" or "comparison group").ti,bt,ab.) and (exp "social isolation"/ or "interpersonal relations"/ or "social interaction"/ or "social adjustment"/ or "social participation"/ or exp "social support"/ or (loneliness or lonely or friendship or solitude or aloneness or "social isolation" or "social participation").ti,ab,kw,bt. or (social adj1 (network* or function* or activ* or interact* or support or contact* or environment* or involv* or connect* or engag* or exclud* or exclus* or relationship* or wellbeing or "well being" or "well-being")).ti. or (social adj2 (network* or function* or activ* or interact* or support or contact* or environment* or involv* or connect* or engag* or relationship* or wellbeing or "well being" or "well-being") adj3 (measure* or promot* or increas* or satisf* or influenc* or improv*)).ti,ab,kw.)) or (exp "Aged"/ and (exp *"social isolation"/ or *"interpersonal relations"/ or *"social interaction"/ or *"social adjustment"/ or *"social participation"/ or exp *"social support"/ or (loneliness or lonely or friendship or solitude or aloneness or "social isolation" or "social participation").ti,bt.) and ("clinical trial".pt. or exp "program evaluation"/ or (intervention* or random* or program* or trial or evaluat* or implement*).ti,bt. or (pilot* or "control group" or "quasi-experimental" or "comparison group").ti,bt,ab.))

4562 results

**DATABASE SEARCHED & TIME PERIOD COVERED**

Cochrane Library (Trials) – limited to January 1, 2011 – June 23, 2021

(([mh "Aging"] or ("older adults" or elder* or senior* or geriatric or aging or ageing or retire* or "old age" or "older people" or "older women" or "older men"):ti,ab,kw) and (exp [mh "program evaluation"] or [mh "epidemiologic studies"] or (intervention* or random* or program* or trial or evaluat* or implement*):ti or (pilot* or "control group" or "quasi-experimental" or "comparison group"):ti,ab) and ([mh "social isolation"] or [mh ^"interpersonal relations"] or [mh ^"social interaction"] or [mh ^"social adjustment"] or [mh ^"social participation"] or [mh "social support"] or (loneliness or lonely or friendship or solitude or aloneness or "social isolation" or "social participation"):ti,ab,kw or (social near/1 (network* or function* or activ* or interact* or support or contact* or environment* or involv* or connect* or engag* or exclud* or exclus* or relationship* or wellbeing or "well being" or "well-being")):ti or (social near/2 (network* or function* or activ* or interact* or support or contact* or environment* or involv* or connect* or engag* or relationship* or wellbeing or "well being" or "well-being") near/3 (measure* or promot* or increas* or satisf* or influenc* or improv*)):ti,ab,kw)) or ([mh "Aged"] and ([mh "social isolation"[mj]] or [mh ^"interpersonal relations"[mj]] or [mh ^"social interaction"[mj]] or [mh ^"social adjustment"[mj]] or [mh ^"social participation"[mj]] or [mh "social support"[mj]] or (loneliness or lonely or friendship or solitude or aloneness or "social isolation" or "social participation"):ti) and ([mh "program evaluation"] or (intervention* or random* or program* or trial or evaluat* or implement*):ti or (pilot* or "control group" or "quasi-experimental" or "comparison group"):ti,ab))

1798 results

**eTable 2. Studies Excluded Due to Data Problems**

| **Study** | **Intervention** | **Data Problem** |
| --- | --- | --- |
| Bickmore, 2005[^76^](#_ENREF_76) | Computer-generated relational agent | Reported only the statistical test of difference between groups, did not report data |
| Davidson, 2014[^32^](#_ENREF_32) | Group singing | Reported only “no differences” in loneliness scores pre- and post-intervention, did not report data |
| Fields, 2019[^80^](#_ENREF_80) | Internet training | No quantitative data reported |
| Fokkema, 2007[^81^](#_ENREF_81) | Various | Does not report sample sizes at the different outcome measurement time points |
| Laron, 2020[^82^](#_ENREF_82) | Various | Did not report data for intervention and comparison groups, only “an average decline of 1.5 points” and then no sample sizes for these data |
| Mullins, 2020[^78^](#_ENREF_78) | Internet training | Reported only “not statistically significant (p=0.13) but the mean total score after the technology classes was lower than the mean at baseline, with a standardized effect size of 0.48” |
| Myhre, 2016[^79^](#_ENREF_79) | Internet training | Only reports “no significant changes”, did not report data |
| Van Rossum, 1993[^75^](#_ENREF_75) | Preventive home visits | Does not report quantitative data, only “no difference with respect to loneliness” |

**eTable 3. Excluded Studies**

Study Design/Qualitative Study, n=26

1. Andrews GJ, G.N., Begley S, Brodie D., Assisting friendships, combating loneliness: users views on a befriending scheme. . Ageing Soc. , 2003. 32: p. 349–62.

2. Baker FA, B.J., You’ve got to accentuate the positive”: group songwriting to promote a life of enjoyment, engagement and meaning in aging Australians. Nord. J Music Ther., 2013. 22: p. 7–24.

3. Ballantyne A., T.L., Zubrinich S. & Corlis M., ‘I feel less lonely’: what older people say about participating in a social networking website. . Quality in Ageing & Older Adults, 2010. 11(3): p. 25-35.

4. Breck, B.M., C.B. Dennis, and S.N. Leedahl, Implementing reverse mentoring to address social isolation among older adults. Journal of gerontological social work, 2018. 61(5): p. 513-525.

5. Butler, S.S., Evaluating the Senior Companion Program: a mixed-method approach. J Gerontol Soc Work, 2006. 47(1-2): p. 45-70.

6. Cant, B. and A. Taket, Promoting social support and social networks among Irish pensioners in South London, UK. Diversity in Health & Social Care, 2005. 2(4): p. 263–270.

7. Cattan, M., N. Kime, and A.M. Bagnall, The use of telephone befriending in low level support for socially isolated older people--an evaluation. Health Soc Care Community, 2011. 19(2): p. 198-206.

8. Cattan M, N.C., Bond J, White M., Alleviating social isolation and loneliness among older people. . Int J Ment Health Promot., 2003. 5: p. 20–30.

9. Cohen-Mansfield J., P.-G.A., Kotler M., Vass J., MacLennan B. & Rosenberg F., Shared interest groups (SHIGs) in low income independent living facilities. Clinical Gerontologist 2007. 31(1): p. 101–112.

10. E., H.A.J., Reducing social isolation and promoting well being in older people. Quality in Ageing & Older Adults 2013. 14(1): p. 25–35.

11. Honigh-de Vlaming R, H.-N.A., Ziylan C, Renes RJ. , Acceptability of the components of a loneliness intervention among elderly Dutch people: a qualitative study. . Am J Heal Educ. , 2013. 44: p. 136–45.

12. Howat P., I.H., Grenade L., Nedwetzky A. & Collins J., Reducing social isolation amongst older people – implications for health professionals. . Geriaction, 2004. 22(1): p. 13–20.

13. Iecovich, E. and A. Biderman, Attendance in adult day care centers and its relation to loneliness among frail older adults. Int Psychogeriatr, 2012. 24(3): p. 439-48.

14. Ito, M., Adler, A., Linde, C., Mynatt, E. and O’Day, V. . Broadening Access : Research for Diverse Network Communities 1998. 1999; Available from: http://www.seniornet.org/research/snaccess_980303.html.

15. Jimenez, F.N., et al., A Technology Training Program to Alleviate Social Isolation and Loneliness Among Homebound Older Adults: A Community Case Study. Front Public Health, 2021. 9: p. 750609.

16. Kime N., C.M.B.A.-M., The delivery and management of telephone befriending services – whose needs are being met? . Quality in Ageing & Older Adults 2012. 13(3): p. 231–240.

17. L., T.M.H., Adult day groups: addressing older people’s needs for activity and companionship. Australasian Journal on Ageing, 2005. 24(3): p. 134–140.

18. Moyle, W., et al., Dementia and loneliness: an Australian perspective. J Clin Nurs, 2011. 20(9-10): p. 1445-53.

19. Pepin, R., et al., Modifying Behavioral Activation to Reduce Social Isolation and Loneliness Among Older Adults. Am J Geriatr Psychiatry, 2021. 29(8): p. 761-770.

20. Pettigrew, S. and M. Roberts, Addressing loneliness in later life. Aging Ment Health, 2008. 12(3): p. 302-9.

21. Savikko N, R.P., Tilvis R, Pitkala K., Psychosocial group rehabilitation for lonely older people: favourable processes and mediating factors of the intervention leading to alleviated loneliness. Int J Older People Nurs., 2009(5): p. 16–24.

22. Stewart M, M.K., Jackson S, et al., Telephone support groups for seniors with disabilities. . Can J Aging, 2001. 20: p. 47–72.

23. Swindell, R., Technology and the over 65s? Get a life. . Social Alternatives, 2001. 20(1): p. 17–23.

24. Swindell R, M.C., Educating the isolated ageing: improving the quality of life of the housebound elderly through educational teleconferencing. Int J Lifelong Educ. , 1996. 15: p. 85–93.

25. Vogelpoel N, J.K., Social prescription and the role of participatory arts programmes for older people with sensory impairments. . J Integr Care., 2014. 22(2): p. 39-50.

26. Wylie, S. Social Isolation and Older People in Canterbury. Age Concern Canterbury, New Zealand. 2012; Available from: http://ageconcerncan.org.nz/resources/

Background/Commentary/Intervention Development/Not Original Research/Protocol, n=12

1. Cohen, B., Research on creativity and aging : the positive impact of the arts on health and illness. . Generations, 2006. 30: p. 7–15.

2. Cyranowski, J.M., et al., Assessing social support, companionship, and distress: National Institute of Health (NIH) Toolbox Adult Social Relationship Scales. Health Psychol, 2013. 32(3): p. 293-301.

3. Dassieu, L. and N. Sourial, Tailoring interventions for social isolation among older persons during the COVID-19 pandemic: challenges and pathways to healthcare equity. International journal for equity in health, 2021. 20(1): p. 26.

4. de Vlaming, R., et al., Evaluation design for a complex intervention program targeting loneliness in non-institutionalized elderly Dutch people. BMC Public Health, 2010. 10: p. 552.

5. Far, I.K., Ferron, M., Ibarra, F., Baez, M., Tranquillini, S., Casati, F., & Doppio, N., The interplay of physical and social wellbeing in older adults: investigating the relationship between physical training and social interactions with virtual social environments. PeerJ Computer Science, 2015. 1(e30).

6. Gale, A., Preventing Social Isolation: A Holistic Approach to Nursing Interventions. Journal of psychosocial nursing and mental health services, 2020. 58(7): p. 11-13.

7. Hughes, M.E., et al., A Short Scale for Measuring Loneliness in Large Surveys: Results From Two Population-Based Studies. Res Aging, 2004. 26(6): p. 655-672.

8. Johnson, J.K., et al., Recruitment and baseline characteristics of the Community of Voices choir study to promote the health and well-being of diverse older adults. Contemp Clin Trials Commun, 2017. 8: p. 106-113.

9. Johnson, J.K., et al., Study protocol for a cluster randomized trial of the Community of Voices choir intervention to promote the health and well-being of diverse older adults. BMC Public Health, 2015. 15: p. 1049.

10. Theeke, L.A. and J.A. Mallow, The Development of LISTEN: A Novel Intervention for Loneliness. Open journal of nursing, 2015. 5(2): p. 136-143.

11. Valtorta, N.K., et al., Loneliness, social isolation and social relationships: what are we measuring? A novel framework for classifying and comparing tools. BMJ Open, 2016. 6(4): p. e010799.

12. Van Orden, K.A., et al., The Senior Connection: design and rationale of a randomized trial of peer companionship to reduce suicide risk in later life. Contemp Clin Trials, 2013. 35(1): p. 117-26.

Population/Setting, n=49

1. Alaviani, M., Khosravan, S., Alami, A., Moshki, M., The Effect of a Multi-strategy Program on Developing Social Behaviors Based on Pender's Health Promotion Model to Prevent Loneliness of Old Women Referred to Gonabad. 2015. 3: p. 132–140.

2. Alaviani, M., et al., The Effect of a Multi-Strategy Program on Developing Social Behaviors Based on Pender's Health Promotion Model to Prevent Loneliness of Old Women Referred to Gonabad Urban Health Centers. International journal of community based nursing and midwifery, 2015. 3(2): p. 132-40.

3. Banks, M.R., L.M. Willoughby, and W.A. Banks, Animal-assisted therapy and loneliness in nursing homes: use of robotic versus living dogs. J Am Med Dir Assoc, 2008. 9(3): p. 173-7.

4. Banks MR, B.W., The effects of animal-assisted therapy on loneliness in an elderly population in long-term care facilities. J Gerontol A Biol Sci Med Sci., 2002. 57: p. M428–M432.

5. Bergman-Evans, B., Beyond the basics. Effects of the Eden Alternative model on quality of life issues. J Gerontol Nurs, 2004. 30(6): p. 27-34.

6. Borji, M. and A. Tarjoman, Investigating the Effect of Religious Intervention on Mental Vitality and Sense of Loneliness Among the Elderly Referring to Community Healthcare Centers. Journal of religion and health, 2020. 59(1): p. 163-172.

7. Brennan, P.F., Moore, S. M. and Smyth, K. A, The effects of a special computer network on care-givers of persons with Alzheimer’s disease. Nursing Research, 1995. 44(3): p. 166–72.

8. Brown, V.M., et al., Indoor gardening older adults: effects on socialization, activities of daily living, and loneliness. J Gerontol Nurs, 2004. 30(10): p. 34-42.

9. Charlesworth, G., et al., Does befriending by trained lay workers improve psychological well-being and quality of life for carers of people with dementia, and at what cost? A randomised controlled trial. Health Technol Assess, 2008. 12(4): p. iii, v-ix, 1-78.

10. Chiang, K.J., et al., The effects of reminiscence therapy on psychological well-being, depression, and loneliness among the institutionalized aged. Int J Geriatr Psychiatry, 2010. 25(4): p. 380-8.

11. Dammeyer, M., Does social isolation among facility-dwelling elderly decrease

using a reminscence group intervention? University of Wyoming, 2004.

12. Drentea, P., et al., Predictors of improvement in social support: Five-year effects of a structured intervention for caregivers of spouses with Alzheimer's disease. Soc Sci Med, 2006. 63(4): p. 957-67.

13. Evans, R.L.a.J., B. M., Phone therapy outreach for blind elderly. The Gerontologist, 1982. 22(1): p. 32–5.

14. Fukui, S., et al., The effect of a psychosocial group intervention on loneliness and social support for Japanese women with primary breast cancer. Oncol Nurs Forum, 2003. 30(5): p. 823-30.

15. Gleibs IH, H.C., Jones JM, et al., No country for old men? The role of a ‘‘gentlemen’s club’’ in promoting social engagement and psychological well-being in residential care. . Aging Ment Health., 2011. 15: p. 456–466.

16. Gustafson, D.H., et al., Pilot Test of a Computer-Based System to Help Family Caregivers of Dementia Patients. J Alzheimers Dis, 2019. 70(2): p. 541-552.

17. Hartke, R.J. and R.B. King, Telephone group intervention for older stroke caregivers. Top Stroke Rehabil, 2003. 9(4): p. 65-81.

18. Jarvis, M.A., A. Padmanabhanunni, and J. Chipps, An Evaluation of a Low-Intensity Cognitive Behavioral Therapy mHealth-Supported Intervention to Reduce Loneliness in Older People. International journal of environmental research and public health, 2019. 16(7).

19. Jones, C.A., et al., Walk, Talk and Listen: a pilot randomised controlled trial targeting functional fitness and loneliness in older adults with hearing loss. BMJ open, 2019. 9(4): p. e026169.

20. Liu S.J., L.C.J., Chen Y.M. & Huang X.Y., The effects of reminiscence group therapy on self-esteem, depression, loneliness and life satisfaction of elderly people living alone. . Mid-Taiwan Journal of Medicine, 2007. 12(3): p. 133–142.

21. Lokk, J., Emotional and social effects of a controlled intervention study in a day-care unit for elderly patients. Scand J Prim Health Care, 1990. 8(3): p. 165-72.

22. Morse, G.A., et al., An experimental comparison of three types of case management for homeless mentally ill persons. Psychiatr Serv, 1997. 48(4): p. 497-503.

23. Morton, T.A., et al., Activating and Guiding the Engagement of Seniors With Online Social Networking: Experimental Findings From the AGES 2.0 Project. J Aging Health, 2018. 30(1): p. 27-51.

24. Moses, B., Technology as a Means of Reducing Loneliness in the Elderly [PhD dissertation]. 2003, Walden University: Minneapolis, Minn.

25. Nikitina, S., et al., Feasibility of Virtual Tablet-Based Group Exercise Among Older Adults in Siberia: Findings From Two Pilot Trials. JMIR mHealth and uHealth, 2018. 6(2): p. e40.

26. O’Loughlin J, L.M., Gagnon G, An evaluation of a volunteer visitor program for socially isolated adults with chronic mental-health problems. [French]. . Canadian Journal of Community Mental Health, 1989. 8: p. 31-52.

27. Pandya, S.P., Meditation program mitigates loneliness and promotes wellbeing, life satisfaction and contentment among retired older adults: a two-year follow-up study in four South Asian cities. Aging & mental health, 2021. 25(2): p. 286-298.

28. Parsons, M., The impact of the Eden alternative on quality of life of nursing home residents. 2004, University of Nebraska.

29. Passmore T, L.D., Tapps T, Gibson H., Impact of participation in community-based recreation program on reported loneliness and feelings of usefulness of individuals diagnoses with early stage Alzheimer’s disease. Am J Recreat Ther., 2007. 6: p. 27–39.

30. Petryshen, P.M., J.D. Hawkins, and T.A. Fronchak, An evaluation of the social recreation component of a community mental health program. Psychiatr Rehabil J, 2001. 24(3): p. 293-8.

31. Reed, M., The mascot model of human/companion interaction: its effects on levels of loneliness and depression among residents of a nursing home. 1986, Western Seminary.

32. Robinson, H., MacDonald, B., Kerse, N., Broadbent, E., The psychosocial effects of a companion robot: a randomized controlled trial. . J. Am. Med. Dir. Assoc., 2013. 14: p. 661–667.

33. Scharlach, A.E., Relieving feelings of strain among women with elderly mothers. Psychology and Aging, 1987. 2(1): p. 9-13.

34. Schulz, R., Effects of control and predictability on the physical and psychological well-being of the institutionalized aged. J Pers Soc Psychol, 1976. 33(5): p. 563-73.

35. Stacey, J., & Edwards, A., Resisting loneliness' dark pit: a narrative therapy approach. Tizard Learning Disability Review., 2013.

36. Theeke, L.A., J.A. Mallow, and E. Theeke, A Pilot One Group Feasibility, Acceptability, and Initial Efficacy Trial of LISTEN for Loneliness in Lonely Stroke Survivors. SAGE open nursing, 2021. 7: p. 23779608211015154.

37. Theurer, K., et al., The development and evaluation of mutual support groups in long-term care homes. J Appl Gerontol, 2014. 33(4): p. 387-415.

38. TM., S., Videoconferencing intervention for depressive symptoms. 2014: Normal: Illinois State University.

39. Toseland, R.W., Rossiter, C. M. and Labrecque, M. S., The effectiveness of peer-led and professionally-led groups to support family caregivers. The Gerontologist, 1989. 29(4): p. 465–71.

40. Toseland, R.W., Long-term effectiveness of peer-led and professionally-led support groups for caregivers. . Social Service Review, 1990. 64: p. 308-27.

41. Toseland, R.W., Rossiter, C. M., Peak, T. and Smith, G. C. , Comparative effectiveness of individual and group interventions to support family caregivers. Social Work, 1990. 35(3): p. 209-17.

42. Travers C, B.H., Silver Memories: implementation and evaluation of a unique radio program for older people. Aging Ment Health, 2010. 15: p. 169–177.

43. Tsai HH, T.Y., Wang HH, et al., Videoconference program enhances social support, loneliness, and depressive status of elderly nursing home residents. . Aging Ment Health. , 2010. 14: p. 947–954.

44. Tsai HH, T.Y., Changes in depressive symptoms, social support, and loneliness over 1 year after a minimum 3-month videoconference program for older nursing home residents. . J Med Internet Res., 2011. 13: p. e93.

45. Tse, M., Therapeutic effects of an indoor gardening programme for older people living in nursing homes. . J Clin Nurs. , 2010. 19: p. 949–958.

46. Tse, M., A. Lo, and T. Cheng, et al., Humor therapy: relieving chronic pain and enhancing happiness for older adults. J Aging Res., 2010. 2010.

47. Vrbanac, Z., et al., Animal assisted therapy and perception of loneliness in geriatric nursing home residents. Coll Antropol, 2013. 37(3): p. 973-6.

48. W.A., B.M.R.B., The effects of group and individual animal-assisted therapy on loneliness in residents of long-term care facilities. Anthrozoos, 2005. 18(4): p. 396–408.

49. Winningham, R. and N. Pike, A cognitive intervention to enhance institutionalized older adults’ social support networks and decrease loneliness. Aging Ment Health., 2007. 11: p. 716–721.

No Intervention, n=14

1. Arsenijevic, J. and W. Groot, Does household help prevent loneliness among the elderly? An evaluation of a policy reform in the Netherlands. BMC public health, 2018. 18(1): p. 1104.

2. Buys, L., Life in a retirement village : implications for contact with community and village friends. . Gerontology, 2001. 47(1): p. 55–61.

3. Cotten, S.R., et al., Internet use and depression among older adults. Computers in human behavior, 2012. 28(2): p. 496-499.

4. Ejiri, M., et al., Social participation reduces isolation among Japanese older people in urban area: A 3-year longitudinal study. PloS one, 2019. 14(9): p. e0222887.

5. Gilbey, A., McNicholas, J., & Collis, G. M., A longitudinal test of the belief that companion animal ownership can help reduce loneliness. Anthrozoös, 2007. 20(4): p. 345-353.

6. Heo, J., et al., Internet use and well-being in older adults. Cyberpsychol Behav Soc Netw, 2015. 18(5): p. 268-72.

7. Hooper, K., Perceptions of loneliness and strategies used by community dwelling older adults who are also part of a seniors’ club to prevent loneliness. 2003, Dalhousie University.

8. Krause-Parello, C.A., Pet ownership and older women: the relationships among loneliness, pet attachment support, human social support, and depressed mood. Geriatr Nurs, 2012. 33(3): p. 194-203.

9. LaVeist, T.A., Sellers, R. M., Brown, K. A. E. and Nickerson, K. J., Extreme social isolation, use of community-based senior support services and mortality among African American elderly women. . American Journal of Community Psychology, 1997. 25(5): p. 721–32.

10. Lee, J. and J.G. Cagle, Validating the 11-Item Revised University of California Los Angeles Scale to Assess Loneliness Among Older Adults: An Evaluation of Factor Structure and Other Measurement Properties. The American journal of geriatric psychiatry : official journal of the American Association for Geriatric Psychiatry, 2017. 25(11): p. 1173-1183.

11. Murayama, Y., et al., The effect of intergenerational programs on the mental health of elderly adults. Aging Ment Health, 2015. 19(4): p. 306-14.

12. Santini, Z.I., et al., Social relationships, loneliness, and mental health among older men and women in Ireland: A prospective community-based study. Journal of affective disorders, 2016. 204: p. 59-69.

13. Smith, J.M., Toward a better understanding of loneliness in community-dwelling older adults. J Psychol, 2012. 146(3): p. 293-311.

14. Toepoel, V., Ageing, Leisure, and Social Connectedness: How could Leisure Help Reduce Social Isolation of Older People? Soc Indic Res, 2013. 113(1): p. 355-372.

Study reports loneliness outcomes, but intervention not aimed at loneliness, n=6

1. Ehlers, D.K., et al., Regional Brain Volumes Moderate, but Do Not Mediate, the Effects of Group-Based Exercise Training on Reductions in Loneliness in Older Adults. Front Aging Neurosci, 2017. 9: p. 110.

2. Mountain, G.A., et al., 'Putting Life in Years' (PLINY) telephone friendship groups research study: pilot randomised controlled trial. Trials, 2014. 15: p. 141.

3. Smith, R., et al., Effect of Group Cognitive Behavioural Therapy on Loneliness in a Community Sample of Older Adults: A Secondary Analysis of a Randomized Controlled Trial. Clinical gerontologist, 2020: p. 1-11.

4. Taube, E., et al., The use of case management for community-dwelling older people: the effects on loneliness, symptoms of depression and life satisfaction in a randomised controlled trial. Scandinavian journal of caring sciences, 2018. 32(2): p. 889-901.

5. Thomas, K.S., U. Akobundu, and D. Dosa, More Than A Meal? A Randomized Control Trial Comparing the Effects of Home-Delivered Meals Programs on Participants' Feelings of Loneliness. The journals of gerontology. Series B, Psychological sciences and social sciences, 2016. 71(6): p. 1049-1058.

6. Wright, L., et al., The Impact of a Home-Delivered Meal Program on Nutritional Risk, Dietary Intake, Food Security, Loneliness, and Social Well-Being. Journal of nutrition in gerontology and geriatrics, 2015. 34(2): p. 218-27.

Study not aimed at loneliness, no loneliness outcomes, n=60

1. Andersson, L., Intervention against loneliness in a group of elderly women: a process evaluation. Human Relations, 1984. 37(4).

2. Arnetz, B.B.a.T., T., Psychological, sociological and health behaviour aspects of a long term activation programme for institutionalized elderly people. Social Science and Medicine, 1983. 17(8): p. 449–56.

3. Bartsch, D.A. and V.K. Rodgers, Senior reach outcomes in comparison with the Spokane Gatekeeper program. Care Manag J, 2009. 10(3): p. 82-8.

4. Bartsch, D.A., V.K. Rodgers, and D. Strong, Outcomes of senior reach gatekeeper referrals: comparison of the Spokane gatekeeper program, Colorado Senior Reach, and Mid-Kansas Senior Outreach. Care Manag J, 2013. 14(1): p. 11-20.

5. Baumgarten, M., et al., Evaluation of a mutual help network for the elderly residents of planned housing. Psychology and Aging, 1988. 3(4): p. 393–8.

6. Bøen, H., Dalgard, O.S., Johansen, R., Nord, E., A randomized controlled trial of a senior centre group programme for increasing social support and preventing depression in elderly people living at home in Norway. . BMC Geriatr., 2012. 12(20).

7. Bogat, G.A.a.J., L. A. , An evaluation of two visiting programs for elderly community residents. International Journal of Aging and Human Development, 1983. 17(4).

8. Broadbent, E., Peri, K., Kerse, N., Jayawardena, C., Kuo, I., Datta, C., & MacDonald, B. , Robots in older people’s homes to improve medication adherence and quality of life: a randomised cross-over trial. In International conference on social robotics 2014. p. 64-73.

9. Caserta, M.S.a.L., D. A., Intrapersonal resources and the effectiveness of self-help groups for bereaved older adults. . The Gerontologist, 1993. 33(5): p. 619-29.

10. Clarke, M., Clarke, S. J. and Jagger, C., Social intervention and the elderly : a randomized controlled trial. American Journal of Epidemiology, 1992. 136(12).

11. Constantino, R.E., Comparison of two group interventions for the bereaved. Image J Nurs Sch, 1988. 20(2): p. 83-7.

12. Conwell, Y., et al., Peer Companionship for Mental Health of Older Adults in Primary Care: A Pragmatic, Nonblinded, Parallel-Group, Randomized Controlled Trial. The American journal of geriatric psychiatry : official journal of the American Association for Geriatric Psychiatry, 2020.

13. De Leo, D., Carollo, G. and Dello Buono, M., Lower suicide rates associated with a Tele-Help/Tele-Check service for the elderly at home. American Journal of Psychiatry, 1995. 152: p. 632–4.

14. Dickens AP, R.S., Hawton A, et al., An evaluation of the effectiveness of a community mentoring service for socially isolated older people: a controlled trial. BMC Public Health, 2011. 11: p. 218.

15. Dubov, A., et al., The physical activity intervention for reducing loneliness in older adults: randomised controlled feasibility trial. European geriatric medicine, 2020. 11(SUPPL 1): p. S230‐.

16. Elderly, C.t., Reaching Isolated Older People. 2007, Contact the Elderly.

17. Fitter, N.T., et al., Exercising with Baxter: preliminary support for assistive social-physical human-robot interaction. Journal of neuroengineering and rehabilitation, 2020. 17(1): p. 19.

18. Florio, E.R., Rockwood, T. H., Hendryx, M. S., Jensen, J. E., Raschko, R. and Dyck, D. G., A model gatekeeper program to find the at-risk elderly. . Journal of Case Management, 1996. 5(3): p. 106–14. .

19. Florio, E.R., Jensen, J. E., Hendryx, M. S., Raschko, R. and Mathieson, K., Oneyear outcomes of older adults referred for aging and mental health services by community gatekeepers. . Journal of Case Management, 1998. 7(2): p. 74–83.

20. Fujiwara Y, S.N., Ohba H, Nishi M, Lee S, Watanabe N, Kousa Y, Yoshida H, Fukaya T, Yajima S, et al., REPRINTS: effects of an intergenerational health promotion program for older adults in Japan. Journal of Intergenerational Relationships, 2009. 7: p. 17-39.

21. Gagliardi, C., et al., A pilot programme evaluation of social farming horticultural and occupational activities for older people in Italy. Health & social care in the community, 2019. 27(1): p. 207-214.

22. Haley, W.E., Brown, S. L. and Levine, E. G., Experimental evaluation of the effectiveness of group intervention for dementia caregivers. The Gerontologist, 1987. 27(3): p. 376–82.

23. Harris JE, B.J., An activity group experience for disengaged elderly persons. Journal of Counseling Psychology, 1978. 25: p. 325-330.

24. Hatamian, A., et. al., Outcomes of the Active at 60 Community Agent Programme. 2012, Department for Work and Pensions.

25. Heyn Billipp, S., The psychosocial impact of interactive computer use within a vulnerable elderly population: a report on a randomized prospective trial in a home health care setting. Public Health Nurs, 2001. 18(2): p. 138-45.

26. Kamegaya, T., et al., Twelve-week physical and leisure activity programme improved cognitive function in community-dwelling elderly subjects: a randomized controlled trial. Psychogeriatrics, 2014. 14(1): p. 47-54.

27. Kapan, A., et al., Impact of a lay-led home-based intervention programme on quality of life in community-dwelling pre-frail and frail older adults: a randomized controlled trial. BMC geriatrics, 2017. 17(1): p. 154.

28. Kim, Y.E. and S.W. Hong, Health-Related Effects of the Elderly Care Program. Biomed Res Int, 2018. 2018: p. 7121037.

29. Knapp, M., et. al., Building community capacity: making an economic case. 2010, PSSRU.

30. Krieger, J., L. Song, and M. Philby, Community health worker home visits for adults with uncontrolled asthma: the HomeBASE Trial randomized clinical trial. JAMA Intern Med, 2015. 175(1): p. 109-17.

31. MacIntyre, I., et al., Pilot study of a visitor volunteer programme for community elderly people receiving home health care. Health Soc Care Community, 1999. 7(3): p. 225-232.

32. McClintock, H.F. and H.R. Bogner, Incorporating Patients' Social Determinants of Health into Hypertension and Depression Care: A Pilot Randomized Controlled Trial. Community Ment Health J, 2017. 53(6): p. 703-710.

33. McEwan, R., Davison, N., Forster, D. P., Pearson, P. and Stirling, E., Screening elderly people in primary care: a randomized controlled trial. . British Journal of General Practice, 1990. 40: p. 99-97.

34. Mulligan, M.A. and R. Bennett, Assessment of mental health and social problems during multiple friendly visits : the development and evaluation of a friendly visiting program for isolated elderly. International Journal of Aging and Human Development, 1977-78. 8: p. 42-65.

35. Mulry, C.M., Piersol, C.V., The Let's go program for community participation: a feasibility study. . Phys. Occup. Ther. Geriatr. , 2014. 32: p. 241–254.

36. Ng, K.S.T., et al., Effects of Horticultural Therapy on Asian Older Adults: A Randomized Controlled Trial. International journal of environmental research and public health, 2018. 15(8).

37. Nicholson, N.R., Shellman, J., Decreasing social isolation in older adults: effects of an empowerment intervention offered through the CARELINK program. . Res. Gerontol. Nurs. , 2013. 6: p. 89-97.

38. Pepin, R., et al., TELE-BEHAVIORAL ACTIVATION FOR SOCIAL ISOLATION IN OLDER HOME-DELIVERED MEALS RECIPIENTS: PRELIMINARY RESULTS FROM AN ONGOING RANDOMIZED CONTROLLED TRIAL. American journal of geriatric psychiatry, 2019. 27(3): p. S129‐S130.

39. Perkins, P., Impact of a horticultural therapy program on the well-being of low-income community dwelling older adults. Acta Hortic., 2012. 954: p. 123–132.

40. Phinney, A., E.M. Moody, and J.A. Small, The Effect of a Community-Engaged Arts Program on Older Adults' Well-being. Can J Aging, 2014. 33(3): p. 336-45.

41. Pitkala, K.H., et al., Effects of psychosocial group rehabilitation on health, use of health care services, and mortality of older persons suffering from loneliness: a randomized, controlled trial. J Gerontol A Biol Sci Med Sci, 2009. 64(7): p. 792-800.

42. Pynoos, J., Hade Kaplan, B. and Fleisher, D., Intergenerational neighborhood networks: a basis for aiding the frail elderly. . The Gerontologist, 1984. 24(3): p. 233–7.

43. Quinn, K., Cognitive effects of social media use: A case of older adults. Social Media+ Society, 2018. 4(3): p. 2056305118787203.

44. Robertson, R.J., Indirect measurement of results in a project for improving socialization among the elderly. . Journal of Gerontology, 1970. 25(3): p. 265–7.

45. Savelkoul, M. and L.P. de Witte, Mutual support groups in rheumatic diseases: Effects and participants' perceptions. Arthritis Rheum, 2004. 51(4): p. 605-8.

46. Schickedanz, A., et al., Impact of Social Needs Navigation on Utilization Among High Utilizers in a Large Integrated Health System: a Quasi-experimental Study. J Gen Intern Med, 2019. 34(11): p. 2382-2389.

47. Seino, S., et al., Effects of a multifactorial intervention comprising resistance exercise, nutritional and psychosocial programs on frailty and functional health in community-dwelling older adults: A randomized, controlled, cross-over trial. Geriatr Gerontol Int, 2017. 17(11): p. 2034-2045.

48. Seino, S., et al., [A 10-year community intervention for disability prevention and changes in physical, nutritional, psychological and social functions among community-dwelling older adults in Kusatsu, Gunma Prefecture, Japan]. [Nihon koshu eisei zasshi] Japanese journal of public health, 2014. 61(6): p. 286-98.

49. Shvedko, A.V., et al., Physical activity intervention for loneliness (PAIL) in community-dwelling older adults: a randomised feasibility study. Psychosomatic medicine, 2019. 81(4): p. A85‐.

50. Song, Y., et al., THE ASSOCIATION BETWEEN A LONELINESS RESOURCE GUIDE AND HEALTH-RELATED QUALITY OF LIFE AMONG A MEDICARE ADVANTAGE POPULATION. American journal of geriatric psychiatry, 2019. 27(3): p. S167‐S168.

51. Tanaka, M., et al., Effect of a human-type communication robot on cognitive function in elderly women living alone. Med Sci Monit, 2012. 18(9): p. CR550-7.

52. Tarazona-Santabalbina, F.J., et al., A Multicomponent Exercise Intervention that Reverses Frailty and Improves Cognition, Emotion, and Social Networking in the Community-Dwelling Frail Elderly: A Randomized Clinical Trial. J Am Med Dir Assoc, 2016. 17(5): p. 426-33.

53. Tesch-Romer, C., Psychological effects of hearing aid use in older adults. Journal of Gerontology : Psychological Sciences, 1997. 52(3): p. P127–38.

54. Toseland, R.W. and G.C. Smith, Effectiveness of individual counseling by professional and peer helpers for family caregivers of the elderly. Psychol Aging, 1990. 5(2): p. 256-63.

55. Trickey, R., N. Kelley-Gillespie, and O.W. Farley, A look at a community coming together to meet the needs of older adults: an evaluation of the neighbors Helping Neighbors program. J Gerontol Soc Work, 2008. 50(3-4): p. 81-98.

56. Tsai, J. and R.A. Rosenheck, Outcomes of a group intensive peer-support model of case management for supported housing. Psychiatr Serv, 2012. 63(12): p. 1186-94.

57. Van Orden, K.A., P.A. Arean, and Y. Conwell, A Pilot Randomized Trial of Engage Psychotherapy to Increase Social Connection and Reduce Suicide Risk in Later Life. The American journal of geriatric psychiatry : official journal of the American Association for Geriatric Psychiatry, 2021.

58. Wikstrom, B., Social interaction associated with visual art discussions: a controlled intervention study. . Aging Ment Health. , 2002. 6: p. 82–87.

59. Yamamoto, S., et al., A Short-Duration Combined Exercise and Education Program to Improve Physical Function and Social Engagement in Community-Dwelling Elderly Adults. International quarterly of community health education, 2020. 40(4): p. 281-287.

60. Yap, A.F., et al., Rhythm-centred music making in community living elderly: a randomized pilot study. BMC Complement Altern Med, 2017. 17(1): p. 311.

Duplicate, n=4

1. Chan, A.W.K., et al., Effects of a peer-assisted tai-chi-qigong programme on social isolation and psychological wellbeing in Chinese hidden elders: a pilot randomised controlled trial. Lancet, 2016. 388(SPEC.ISS 1): p. 23‐.

2. Jones, R.B., et al., Older people going online: its value and before-after evaluation of volunteer support. Journal of medical Internet research, 2015. 17(5): p. e122.

3. Moieni, M., et al., Feeling needed: Effects of a randomized generativity intervention on well-being and inflammation in older women. Brain, behavior, and immunity, 2020. 84: p. 97-105.

4. Stevens NL, M.C., Westerhof GJ., Meeting the need to belong: predicting effects of a friendship enrichment program for older women. . Gerontologist., 2006. 46: p. 495–502.

Unavailable, n=3

1. K., C.M.I., Implementing change: the alleviation of social isolation and loneliness among older people in Leeds. Journal of Mental Health Promotion 2003. 2(3): p. 12–19.

2. P., W., The effects of animal-assisted therapy on loneliness in elderly residents of a long-term care facility utilizing Roy’s adaptation model:. 1995, University of Southern Mississippi.

3. Shanley, C., Teleconferencing as a strategy for carer support. Australasian Journal on Ageing, 2001. 20(3).

Does Not Use UCLA or de Jong Scale, n=14

1. Blazun H., S.K.R.S., Impact of computer training courses on reduction of loneliness of older people in Finland and Slovenia. . Computers in Human Behavior 2012. 28: p. 1202–1212.

2. Bruce, M.L., et al., One Year Impact on Social Connectedness for Homebound Older Adults: Randomized Controlled Trial of Tele-delivered Behavioral Activation Versus Tele-delivered Friendly Visits. The American journal of geriatric psychiatry : official journal of the American Association for Geriatric Psychiatry, 2021.

3. Choi, N.G., et al., Improving Social Connectedness for Homebound Older Adults: Randomized Controlled Trial of Tele-Delivered Behavioral Activation Versus Tele-Delivered Friendly Visits. The American journal of geriatric psychiatry : official journal of the American Association for Geriatric Psychiatry, 2020. 28(7): p. 698-708.

4. Cox EO, G.K., Hobart K, et al., Strengthening the late-life care process: effects of two forms of a care-receiver efficacy intervention. Gerontologist. , 2007. 47: p. 388-397.

5. Fields, J., et al., In-Home Technology Training Among Socially Isolated Older Adults: Findings From the Tech Allies Program. Journal of applied gerontology : the official journal of the Southern Gerontological Society, 2021. 40(5): p. 489-499.

6. Gustafsson, S., et al., Minor positive effects of health-promoting senior meetings for older community-dwelling persons on loneliness, social network, and social support. Clinical interventions in aging, 2017. 12: p. 1867-1877.

7. Hopman-Rock, M.a.W., M. H., Development and evaluation of ‘Aging well and healthily ’ : a health education and exercise program for community living older adults. . Journal of Aging and Physical Activity, 2002. 10.

8. Morrow-Howell, N., Becker-Kemppainen, S. and Lee, J., Evaluating an intervention for the elderly at increased risk of suicide. . Research on Social Work Practice, 1998. 8(1): p. 28-46.

9. Ollonqvist K, P.H., Aaltonen T, et al. , Alleviating loneliness among frail older people—findings from a randomised controlled trial. . Int J Ment Health Promot., 2008. 10: p. 26–34.

10. Pynnonen, K., et al., Effect of a social intervention of choice vs. control on depressive symptoms, melancholy, feeling of loneliness, and perceived togetherness in older Finnish people: a randomized controlled trial. Aging Ment Health, 2018. 22(1): p. 77-84.

11. Rosen, C.E.a.R., S. , Evaluating an intervention program for the elderly. Community Mental Health Journal, 1982. 18(1): p. 21-33.

12. Sorensen, K.H.a.S., J., Follow-up three years after intervention to relieve unmet medical and social needs of old people. . Comprehensive Gerontology : Section B, Behavioural, Social and Applied Sciences,, 1988. 2(2): p. 85-91.

13. Stewart, M., Craig, D., MacPherson, K. and Alexander, S., Promoting positive affect and diminishing loneliness of widowed seniors through a support intervention. Public Health Nursing, 2001. 18(1): p. 54-63.

14. Theunissen, I., Spinhoven, P. and van der Does, Omgaan met alleenstaan : Evaluatie van een groepscursus voor ouderen weduwen [Coping with loneliness : evaluation of a group course for elderly widows]. . Tijdschrift voor Gerontologie en Geriatrie, 1994. 25(6): p. 250–4.

No Usable Data, n=8

1. Bickmore TW, C.L., Clough-Gorr K, Heeren T. , ‘It’s just like you talk to a friend’’ relational agents for older adults. Interact Comput., 2005. 17: p. 711-735.

2. Davidson, J.W., McNamara, B., Rosenwax, L., Lange, A., Jenkins, S., Lewin, G., Evaluating the potential of group singing to enhance the well-being of older people. Australas. J. Ageing 2014. 33: p. 99-104.

3. Fields, J., et al., In-home technology training to reduce social isolation and improve tablet use among older adults: findings from the tech allies program. Journal of general internal medicine, 2019. 34(2): p. S258‐S259.

4. Fokkema, C.M. and T.G. van Tilburg, [Loneliness interventions among older adults: sense or nonsense?]. Tijdschr Gerontol Geriatr, 2007. 38(4): p. 185-203.

5. Laron, M.M., Ittay & Cohen, Yafit & Weiss, Dana & Kagya, Shlomit., The Project to Reduce and Cope with Feelings of Loneliness among Older Adults A Formative Evaluation. . 2020.

6. Mullins, L.B., et al., Internet Programming to Reduce Loneliness and Social Isolation in Aging. Research in gerontological nursing, 2020. 13(5): p. 233-242.

7. Myhre, J.W., M.R. Mehl, and E.L. Glisky, Cognitive Benefits of Online Social Networking for Healthy Older Adults. Journals of gerontology. Series B, Psychological sciences and social sciences, 2017. 72(5): p. 752‐760.

8. van Rossum, E., et al., Effects of preventive home visits to elderly people. British Medical Journal, 1993. 307: p. 27–32.

Short Follow-up Time, n=5

1. Bartlett, M.Y. and S.N. Arpin, Gratitude and Loneliness: Enhancing Health and Well-Being in Older Adults. Research on aging, 2019. 41(8): p. 772-793.

2. Gaggioli, A., Morganti, L., Bonfiglio, S., Scaratti, C., Cipresso, P., Serino, S., Riva, G. , Intergenerational group reminiscence: a potentially effective intervention to enhance elderly psychosocial wellbeing and to improve Children's perception of aging. . Educ. Gerontol., 2014. 40: p. 486–498.

3. Hansen, P., C. Main, and L. Hartling, Dance Intervention Affects Social Connections and Body Appreciation Among Older Adults in the Long Term Despite COVID-19 Social Isolation: A Mixed Methods Pilot Study. Frontiers in psychology, 2021. 12: p. 635938.

4. Sidner CL, B.T., Nooraie B, et al., Creating new technologies for companionable agents to support isolated older adults. ACM Trans Interact Intell Syst. , 2018. 8(3): p. 1-27.

5. Wang, D.S., Feasibility of a Yoga Intervention for Enhancing the Mental Well-Being and Physical Functioning of Older Adults Living in the Community, Activities, Adaptation & Aging. 2010. 34(2): p. 85-97.

Study Quality, n=1

1. Foster, A., et al., Impact of social prescribing to address loneliness: A mixed methods evaluation of a national social prescribing programme. Health Soc Care Community, 2021. 29(5): p. 1439-1449.

| **eTable 4. Risk of Bias for RCTs (Cochrane Risk of Bias)** | | | | | | |
| --- | --- | --- | --- | --- | --- | --- |
| **Author, Year** | **Random** | **Allocation Concealment** | **Blinding Participants** | **Blinding Outcome Assessment** | **Selective Reporting** | **Attrition** |
| White, 2002[^105^](#_ENREF_105) | ⯋ | ⯋ | ● | ● | ○ | ○ |
| Rook, 2003 [^113^](#_ENREF_113) | ⯋ | ⯋ | ● | ● | ○ | ● |
| de Craen, 2006[^114^](#_ENREF_114) | ○ | ○ | ● | ● | ○ | ● |
| Kremers, 2006[^43^](#_ENREF_43) | ⯋ | ⯋ | ● | ● | ○ | ● |
| Routasalo, 2009[^90^](#_ENREF_90) | ○ | ○ | ● | ● | ○ | ⯋ |
| Slegers, 2008[^99^](#_ENREF_99) | ⯋ | ⯋ | ● | ● | ○ | ○ |
| Hind, 2014[^115^](#_ENREF_115) | ○ | ○ | ● | ● | ○ | ● |
| Saito, 2012[^42^](#_ENREF_42) | ⯋ | ⯋ | ● | ● | ○ | ○ |
| Hall, 1992[^116^](#_ENREF_116) | ○ | ⯋ | ● | ● | ○ | ○ |
| Chan, 2017[^45^](#_ENREF_45) | ○ | ○ | ● | ● | ○ | ● |
| Mountain, 2017[^88^](#_ENREF_88) | ○ | ○ | ● | ● | ○ | ○ |
| Creswell, 2012[^89^](#_ENREF_89) | ○ | ⯋ | ● | ● | ○ | ○ |
| Kahlon, 2021[^117^](#_ENREF_117) | ○ | ○ | ● | ● | ○ | ○ |
| Boekhout, 2021[^118^](#_ENREF_118) | ○ | ○ | ● | ● | ○ | ○ |
| Larsson, 2016[^107^](#_ENREF_107) | ○ | ○ | ● | ● | ○ | ○ |
| Rodriguez-Romero, 2021[^86^](#_ENREF_86) | ⯋ | ⯋ | ● | ● | ○ | ○ |
| Shapira, 2021[^104^](#_ENREF_104) | ○ | ○ | ● | ● | ○ | ● |
| Lai, 2020[^119^](#_ENREF_119) | ● | ● | ● | ● | ○ | ○ |
| Rolandi, 2020[^96^](#_ENREF_96) | ⯋ | ⯋ | ● | ● | ○ | ○ |
| Weiss, 2020[^120^](#_ENREF_120) | ○ | ○ | ● | ● | ○ | ○ |
| Ristolainen, 2020[^41^](#_ENREF_41) | ⯋ | ⯋ | ● | ● | ○ | ○ |
| Haslam, 2019[^84^](#_ENREF_84) | ○ | ⯋ | ● | ● | ○ | ● |
| Brodbeck, 2019[^106^](#_ENREF_106) | ○ | ○ | ● | ● | ○ | ○ |
| Cohen-Mansfield, 2018[^121^](#_ENREF_121) | ⯋ | ⯋ | ● | ● | ○ | ● |
| Czaja, 2018[^97^](#_ENREF_97) | ⯋ | ⯋ | ● | ● | ○ | ○ |
| Hill, 2006[^85^](#_ENREF_85) | ⯋ | ⯋ | ● | ● | ○ | ○ |
| Woodward, 2011[^98^](#_ENREF_98) | ⯋ | ⯋ | ● | ● | ○ | ● |
| Kall, 2020[^40^](#_ENREF_40) | ○ | ○ | ● | ● | ○ | ○ |
| Kall, 2021[^39^](#_ENREF_39) | ○ | ○ | ● | ● | ○ | ● |
| Dodge, 2015[^109^](#_ENREF_109) | ⯋ | ● | ● | ● | ○ | ○ |
| Baez, 2017[^108^](#_ENREF_108) | ○ | ○ | ● | ● | ○ | ○ |
| Chow, 2019[^92^](#_ENREF_92) | ○ | ● | ● | ○ | ○ | ● |
| Theeke, 2016[^91^](#_ENREF_91) | ⯋ | ● | ● | ● | ○ | ○ |
| Johnson, 2020[^31^](#_ENREF_31) | ○ | ○ | ● | ● | ○ | ○ |
| Moieni, 2021[^122^](#_ENREF_122) | ⯋ | ● | ● | ○ | ○ | ○ |
| Heller, 1991[^30^](#_ENREF_30) | ● | ⯋ | ● | ● | ○ | ● |

○ = Low Risk of Bias, ● = High Risk of Bias, ⯋ = Unclear

**eTable 5. Risk of Bias in Non-randomized Studies of Interventions (ROBINS-I)**

| **Author, Year** | **Confounding** | **Selection bias** | **Bias in measurement classification of interventions** | **Bias due to deviations from intended interventions** | **Bias due to missing data** | **Bias in measurement of outcomes** | **Bias in selection of the reported result** |
| --- | --- | --- | --- | --- | --- | --- | --- |
| White, 1999[^105^](#_ENREF_105) | High | High | Low | Low | Low | Low | Low |
| Cohen, 2006[^44^](#_ENREF_44) | High | High | Low | Low | Low | Low | Low |
| Martina, 2006[^36^](#_ENREF_36) | High | High | Low | Low | Low | Low | Low |
| Shapira, 2007[^104^](#_ENREF_104) | High | High | Low | Low | Low | Low | Low |
| Honigh, 2013[^123^](#_ENREF_123) | High | High | Low | Low | High | Low | Low |
| Stevens, 2000[^38^](#_ENREF_38) | Low | High | Low | Low | High | Low | Low |
| Winstead, 2014 [^124^](#_ENREF_124) | High | High | Low | High | High | Low | Low |
| Van den Elzen, 2006[^125^](#_ENREF_125) | Low | High | Low | Low | High | Low | Low |

**eTable 6. Risk of Bias in Pre-Post studies**

| **Author, Year** | **Were eligibility/selection criteria for the study population prespecified and clearly described?** | **Was the sample size sufficiently large to provide confidence in the findings?** | **Was the test/service/ intervention clearly described and delivered consistently across the study population?** | **Were the outcome measures prespecified, clearly defined, valid, reliable, and assessed consistently across all study participants?** | **Was the loss to follow-up after baseline 20% or less? Were those lost to follow-up accounted for in the analysis?** |
| --- | --- | --- | --- | --- | --- |
| Stevens, 2001[^37^](#_ENREF_37) | Yes | No | Yes | Yes | No |
| Collins, 2006[^94^](#_ENREF_94) | Yes | Yes | Yes | Yes | Yes |
| Fokkema, 2007[^103^](#_ENREF_103) | Yes | No | Yes | Yes | No |
| Bartlett, 2013[^126^](#_ENREF_126) | Yes | No | Yes | Yes | Yes |
| Jones, 2015[^102^](#_ENREF_102) | Yes | Yes | Yes | Yes | Yes |
| Low, 2015[^127^](#_ENREF_127) | Yes | Yes | Yes | Yes | No |
| Van Der Heide, 2012[^128^](#_ENREF_128) | Yes | Yes | Yes | Yes | No |
| Coll-Planas, 2017[^93^](#_ENREF_93) | Yes | No | Yes | Yes | No |
| Gonyea, 2013[^129^](#_ENREF_129) | Yes | No | Yes | Yes | Yes |
| Juang, 2020[^130^](#_ENREF_130) | Yes | No | Yes | Yes | No |
| Neil-Sztramko, 2020[^101^](#_ENREF_101) | Yes | No | Yes | Yes | Yes |
| Roberts, 2020[^131^](#_ENREF_131) | Yes | Yes | Yes | Yes | No |
| Levinger, 2020[^47^](#_ENREF_47) | Yes | Yes | Yes | Yes | No |
| McKay, 2018[^95^](#_ENREF_95) | Yes | Yes | Yes | Yes | Yes |
| McAuley, 2000[^46^](#_ENREF_46) | Yes | Yes | Yes | Yes | Yes |
| Vella-Burrows, 2021[^132^](#_ENREF_132) | No | No | No | Yes | No |

**eTable 7. Details of Internet-Delivered Intervention Studies**

| **Study/Country/Study design** | **Age of subjects** | **Selected for increased loneliness risk?** | **Type of therapy** | **Duration** |
| --- | --- | --- | --- | --- |
| Baez, 2016[^108^](#_ENREF_108)  Italy  **RCT** | Mean age = 71 | No | Online group exercises | 8 weeks of exercises followed by 1 week of post-exercise measurements |
| Dodge, 2015[^109^](#_ENREF_109)  USA  **RCT** | Mean age = 81 | No | Videochat with trained interviewers using a conversational protocol | Conversations with trained interviewers 5 days a week for 6 weeks |
| Brodbeck, 2019[^106^](#_ENREF_106)  Switzerland  **RCT** | Mean age = 51 | Yes | CBT^1^ for spousal bereavement | 10 sessions over 10 weeks; 24 week follow-up |
| Hill, 2006[^85^](#_ENREF_85)  USA  **RCT** | Ages 35-65 | Yes | “Women-to-women project” – support group and health teaching units | 22 weeks |
| Kall, 2020[^40^](#_ENREF_40)  Sweden  **RCT** | Mean age = 47.2 | Yes | Internet CBT | 8 modules over 8 weeks |
| Kall, 2021[^39^](#_ENREF_39)  Sweden  **RCT** | Mean age = 47.5 | Yes | Internet CBT  Internet Interpersonal psychotherapy | 9 modules over 8 weeks; 10 week follow-up |
| Larsson, 2016[^107^](#_ENREF_107)  Sweden  **RCT** | > 60 | Yes | Social-internet based activities based on the Occupational Therapy Intervention Process Model, individual and group meeting | Not reported |

^1^Cognitive Behavioral Therapy

**eTable 8. Details of Individual In-Person Contact Interventions**

| **Study/Country/Study design** | **Age of subjects** | **Selected for increased loneliness risk?** | **Type and content of contact** | **Duration** |
| --- | --- | --- | --- | --- |
| de Craen, 2006[^114^](#_ENREF_114)  Netherlands  **RCT** | Mean age = 85 | Yes | Home visit by an occupational therapist | One visit, with 24 month follow-up |
| Hall, 1992[^116^](#_ENREF_116)  Canada  **RCT** | > 65 | Yes | “Frail Elders Personalized Program” – a series of home visits by a nurse to assess and suggest treatment interventions for health care, substance use, exercise, nutrition, stress management, emotional functioning, social support and participation, housing, finances, and transportation | Multiple home visits over many months; 36 month follow-up |
| Lai, 2020[^119^](#_ENREF_119)  Canada  **RCT** | > 65 | Yes | Peer-support services through home visits, phone calls, and activities such as emotional support, referrals, help to establish goals such as self-care and social engagement, problem solving, and mental health and community resources | 8 week intervention; 10 week follow-up |
| Roberts, 2020[^131^](#_ENREF_131)  Wales  **Pre-post** | > 50 | Yes | A one-on-one interaction with a volunteer that offered the subject companionship, practical and psychological support, and to increase social networks, confidence, and independence | Not reported |
| van den Elzen, 2006[^125^](#_ENREF_125)  Netherlands  **Pre-post** | Age = “elderly” | Yes | Home visits to contact lonely people and identify needs and wishes regarding welfare services | 6-12 month follow-up |
| Weiss, 2020[^120^](#_ENREF_120)  Netherlands  **RCT** | Median age = 60 | Yes | “The Happiness Route” – a positive psychology intervention using the principles of self-determination, delivered by a counselor making home visits | 3 months |

**eTable 9. Details of Telephone-Based Interventions**

| **Study/Country/Study Design** | **Age of subjects** | **Selected for increased loneliness risk?** | **Type of remote-based contact** | **Duration** |
| --- | --- | --- | --- | --- |
| Heller, 1991[^30^](#_ENREF_30)  USA  **RCT** | Median age = 74 | Yes | Periodic telephone contacts from trained interviewers, discussing health, well-being, and events in their lives | Twice a week for 5 weeks, then once per week for 5 weeks |
| Hind, 2014[^115^](#_ENREF_115)  England  **RCT** | > 75 | Yes | “Telephone friendship” – one-to-one befriending by telephone, and teleconference groups of up to 6 participants, aimed to enhance social support and increase opportunities for social interaction | Once per week for 6 weeks, groups every 2 weeks; 6 month follow-up |
| Juang, 2020[^130^](#_ENREF_130)  USA  **Pre-post** | > 65 | No | RESOLV – a telephone-based socialization program for older Veterans. | 6 months |
| Kahlon, 2021[^117^](#_ENREF_117)  USA  **RCT** | Mean age = 69 | Yes | Laypeople engaging regularly with empathetic intention through telephone calls with participants | 1. weeks of phone calls |

**eTable 10. Details of Miscellaneous Studies**

| **Study/Country/Study design** | **Age of subjects** | **Selected for increased loneliness risk?** | **Type and content of contact** | **Duration** |
| --- | --- | --- | --- | --- |
| Boekhout, 2021[^118^](#_ENREF_118)  Netherlands  **RCT** | > 65 | Yes | Active Plus – a computer -tailored intervention designed to stimulate cognitive function and physical activity and decrease loneliness | 12 months |
| Cohen-Mansfield, 2018[^121^](#_ENREF_121)  Israel  **RCT** | > 65 | Yes | I-SOCIAL – an intervention focused on addressing psychosocial barriers and environmental barriers. Identifying individual barriers, then up to 10 individual meetings with a counselor; up to 7 group sessions to increase social competence. | Duration of intervention varies; 3 month follow-up |
| Moieni, 2021[^122^](#_ENREF_122)  USA  **RCT** | Mean age = 71 | Yes | Writing alone about sharing their life experiences with the next generation | 6 weekly sessions |
| Rook, 2003[^113^](#_ENREF_113)  USA  **RCT** | Mean age = 70 | No | Volunteer as a Foster Grandparent to a developmentally-disabled child in residence at a state hospital | 1-2 years |
| Low, 2015[^127^](#_ENREF_127)  Australia  **Pre-post** | Mean age = 83 | Yes | Lifestyle Engagement Activity Program - training home care workers to help assess the individualized needs of home care clients for the frequency and type of social support needed, which might include teaching computer skills to transport assistance to making traditional foods to going to the library | 12 months |
| Gonyea, 2013[^129^](#_ENREF_129)  USA  **Pre-post** | > 70 | No | Aging Well at Home – the concept of a Village to help older adults stay at home, which includes a community liaison, safe houses, and community forums. | 9 months |
| Honigh, 2013[^123^](#_ENREF_123)  Netherlands  **Non-randomized study** | Mean age = 74 | Yes | Healthy Aging – a complex intervention including a mass media campaign, information meetings, psychosocial group courses, and social activities organized be neighbors | 2 years |
| Van der Heide, 2012[^128^](#_ENREF_128)  Netherlands  **Pre-post** | Median age = 73 | No | CareTV – 24/7 access to communicate with a nurse via video and voice connection. It can also be used to contact family and friends. | Not reported precisely, but likely many months |

**eTable 11. Details of Group Activities Studies**

| **Study/Country/Study design** | **Age of subjects** | **Selected for increased loneliness risk?** | **Details of activity** | **Duration of activity** |
| --- | --- | --- | --- | --- |
| Bartlett, 2013[^126^](#_ENREF_126)  Australia  **Pre-post** | Mean age = 83 | No | Various activities such as exercises, arts programs, “leisure activities” plus other potential components like a “buddy system” | Not reported |
| Cohen, 2006[^44^](#_ENREF_44)  USA  **Non-randomized study** | > 64 | No | Singing in a chorus | 30 weeks; 12 month follow-up |
| Winstead, 2014[^124^](#_ENREF_124)  USA  **Non-randomized study** | Mean age = 80 | No | Various activities | 90 minutes twice a week for 8 weeks; 3 month followup |
| Johnson, 2018[^31^](#_ENREF_31)  USA  **Randomized study** | Mean age = 71 | No | Singing in a choir | 90 minutes weekly for 44 weeks |
| Vella-Burrows, 2021[^132^](#_ENREF_132)  England  **Pre-post** | Mean age = “older population” | No | Dance to Health, which integrates physiotherapy with artistic dance | 90 minute dance sessions twice a week for 6 months |

eTable 12. Studies Excluded because they did not use the UCLA scale or the deJong Gierveld scale

We excluded 14 studies because they did use the UCLA scale or the deJong Gierveld scale.

8 studies reporting dichotomous outcomes:

Group-based treatments

Gustafsson 2017

Rosen 1982

Internet training

Fields 2021 – note this study actually used the UCLA scale but reported the outcome as dichotomous into “lonely” and “not lonely”

Blazun 2012 – no odds ratio able to be calculated

Miscellaneous

GoodMood – Pyonnen 2018

Social worker and physician visit – Sorenson 1988

Theunisen – unable to calculate an OR

Group inpatient rehabilitation in community living frail adults – Ollonqvist 2008

For the studies for which we were able to calculate an odds ratio, they are presented in a Forest plot labeled eTable 12.1

6 studies reporting scales:

Telephone-delivered interventions

Bruce 2021

Choi 2020

Group based treatments

Cox 2007

Stewart 2001

Miscellaneous

Group meetings + group exercise – Hopman-Rock, 2002

LifeCrisis – Morrow-Howell 1998

These 6 studies are presented in a Forest plot labeled eTable 12.2

eTable 12.1


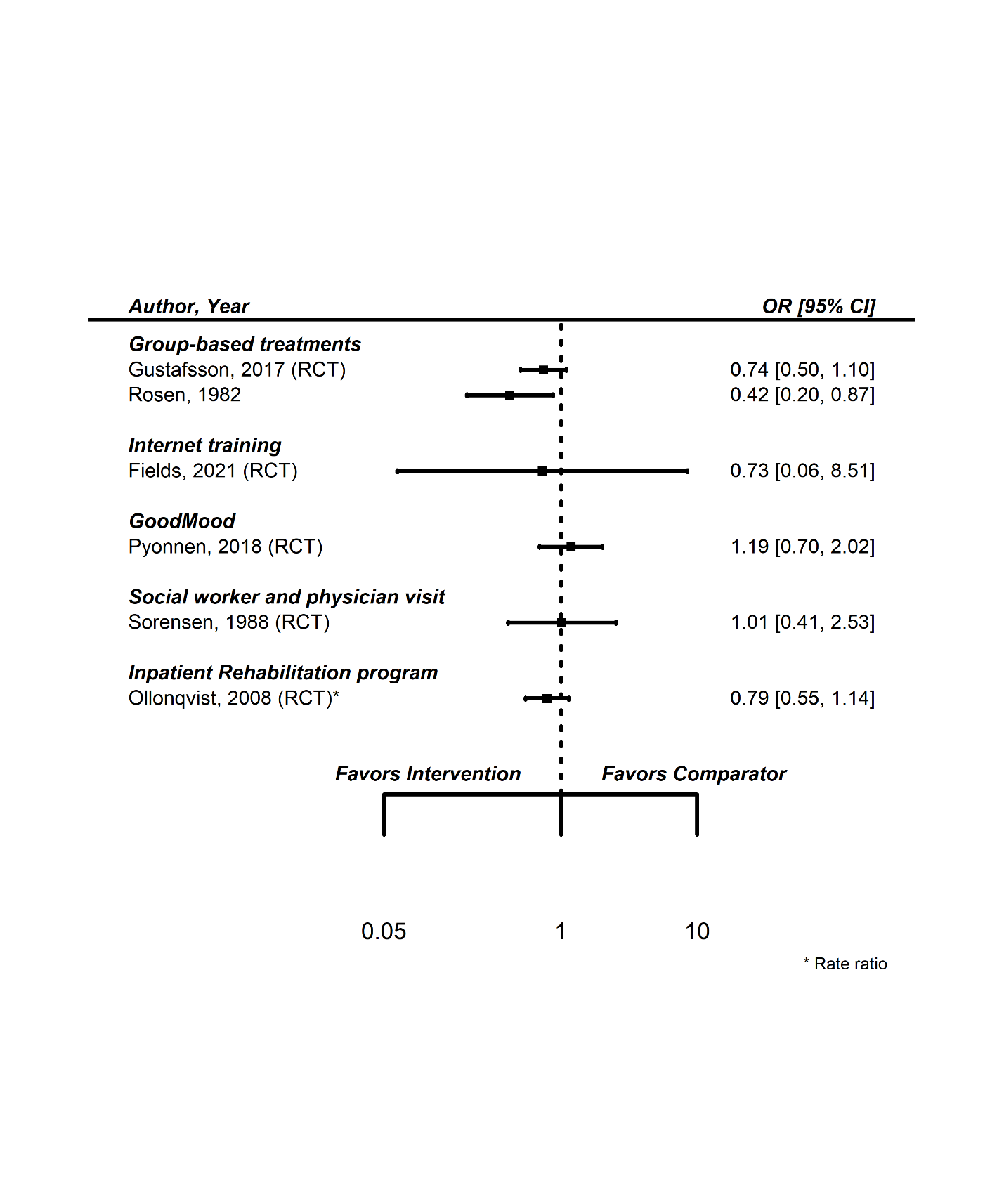


eTable 12.2


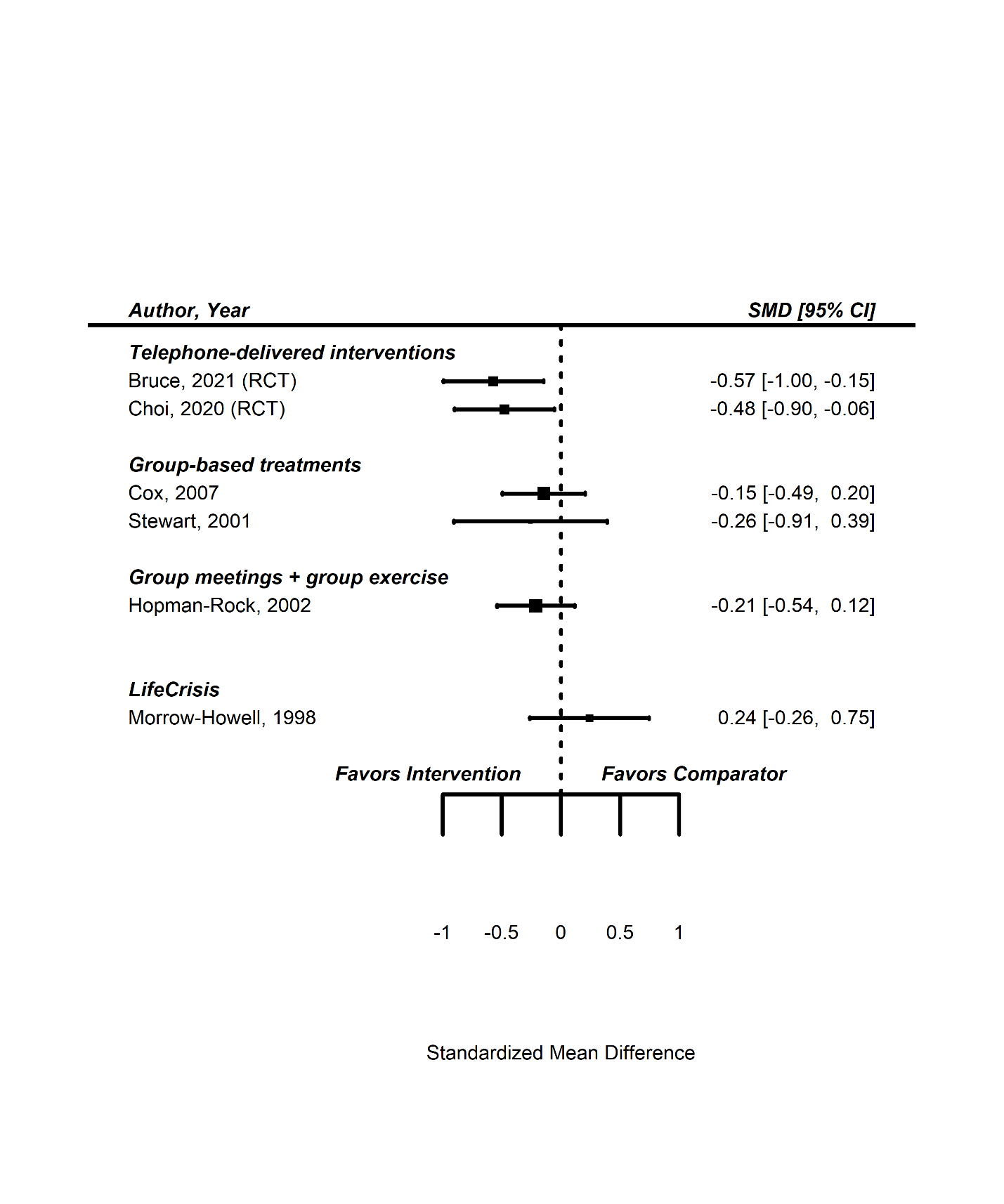


eTable 13. GRADE Certainty of Evidence Table

| **Intervention** | **Study Limitations** | **Consistency** | **Directness** | **Precision** | **Certainty of Evidence** |
| --- | --- | --- | --- | --- | --- |
|  |  |  |  |  |  |
| Group-based treatments are associated with less loneliness | No serious limitations | Serious inconsistency | No serious indirectness | No serious imprecision | Moderate |
| Group-based exercises are associated with less loneliness | Serious limitations | No serious inconsistency | No serious indirectness | Serious imprecision | Low |
| Internet-training is associated with less loneliness | No serious limitation | Serious inconsistency | No serious indirectness | No serious imprecision | Moderate |
| Internet-delivered interventions are associated with less loneliness | Serious limitation | No serious inconsistency | Serious indirectness | No serious imprecision | Very Low |

**eTable 14. Comparison of Included Studies in Hoang Review and this Review**

The meta-analysis by Hoang and colleagues^[[1]](#footnote-1)^ included 27 RCTs of interventions for community living older adults. The 13 RCTs that met our inclusion criteria are included in our analysis.[^43^](#_ENREF_43)^,^[^45^](#_ENREF_45)^,^[^88^](#_ENREF_88)^,^[^90-92^](#_ENREF_90)^,^[^97^](#_ENREF_97)^,^[^99^](#_ENREF_99)^,^[^107-109^](#_ENREF_107)^,^[^113^](#_ENREF_113)^,^[^121^](#_ENREF_121) The remainder were excluded for the following reasons: 6 focused on a specific population (cancer,[^133^](#_ENREF_133) hearing loss,[^134^](#_ENREF_134) caregivers for person with dementia,[^135^](#_ENREF_135)^,^[^136^](#_ENREF_136) caregivers to persons with stroke,[^137^](#_ENREF_137), residential care[^138^](#_ENREF_138)), 1 from a LMIC,[^26^](#_ENREF_26) 2 follow-up time too short,[^73^](#_ENREF_73)^,^[^74^](#_ENREF_74) 2 studies not aimed at loneliness,[^139^](#_ENREF_139)^,^[^140^](#_ENREF_140) 1 pilot study,[^141^](#_ENREF_141) 2 no useable data.[^76^](#_ENREF_76)^,^[^142^](#_ENREF_142)

The Hoang and colleagues review included in pooled analyses populations we excluded, including persons who all had specific health conditions, such as cancer,[^133^](#_ENREF_133) or caregivers for persons with dementia;[^135^](#_ENREF_135) studies in Low and Middle Income Countries;[^26^](#_ENREF_26) and studies whose primary purpose we judged to not be reducing loneliness. Other differences included their restriction to RCTs only, that our search was more recent and thus includes a dozen newer RCTs, including some pandemic-era studies; and some of the details of the meta-analytic methods. However, perhaps the biggest difference between the two reviews is how studies were grouped for analysis. In the review by Hoang and colleagues the interventions were grouped into broad categories: counseling, therapy, exercise, social interventions, and technological interventions. Our groupings were different, and considered both how the intervention was delivered and its content.

**eFigure 1. Literature Flow**

#
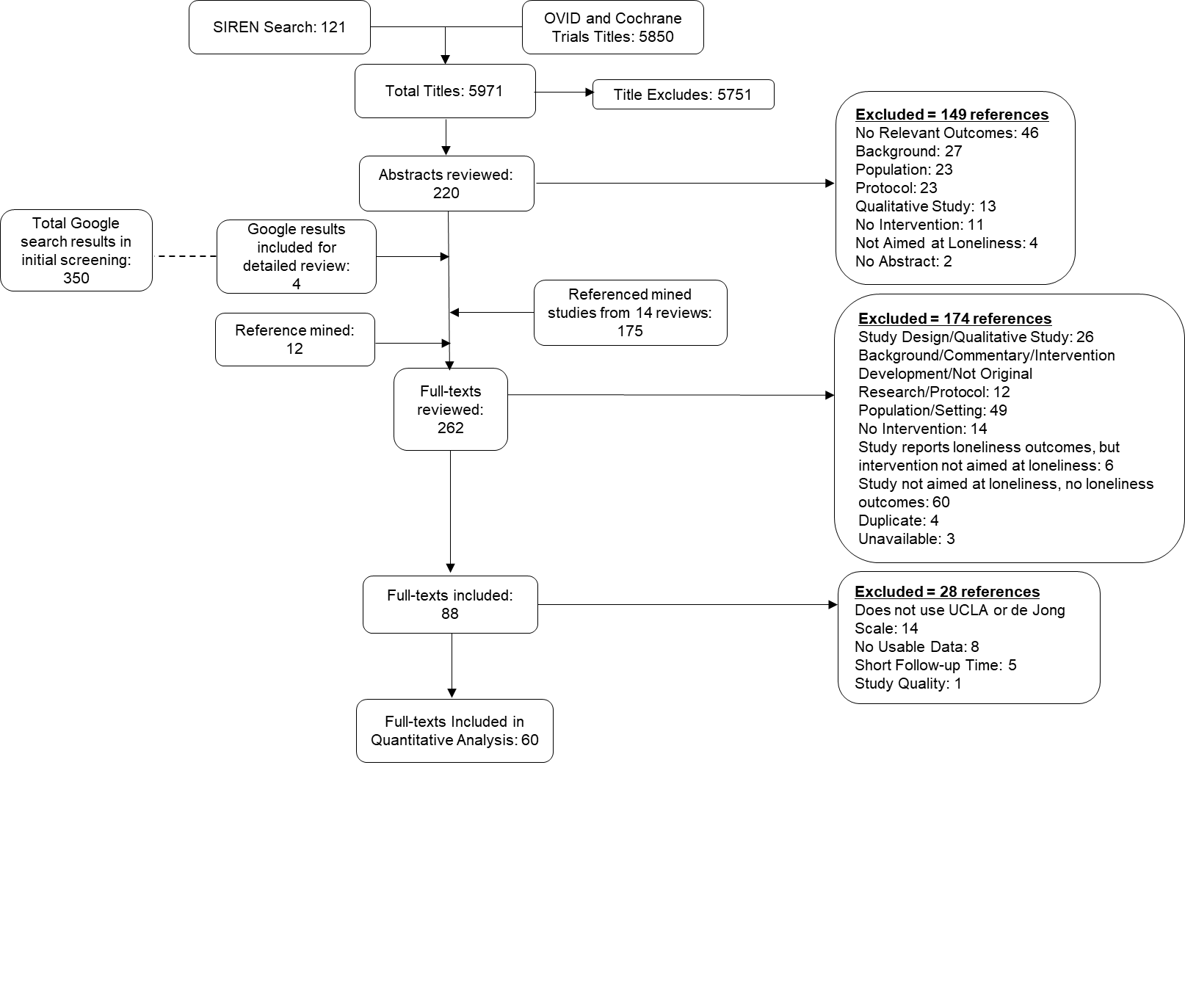


1. Hoang P, King JA, Moore S, Moore K, Reich K, Sidhu H, et al. Interventions Associated With Reduced Loneliness and Social Isolation in Older Adults: A Systematic Review and Meta-analysis. JAMA Netw Open. 2022;5(10):e2236676. [↑](#footnote-ref-1)
